# Integrative Migraine Therapy: From Current Concepts to Future Directions—A Plastic Surgeon’s Perspective

**DOI:** 10.3390/medicina62010050

**Published:** 2025-12-26

**Authors:** Cristian-Sorin Hariga, Eliza-Maria Bordeanu-Diaconescu, Andrei Cretu, Dragos-Constantin Lunca, Catalina-Stefania Dumitru, Cristian-Vladimir Vancea, Florin-Vlad Hodea, Stefan Cacior, Vladut-Alin Ratoiu, Andreea Grosu-Bularda

**Affiliations:** 1 Department 11, Discipline Plastic and Reconstructive Surgery, University of Medicine and Pharmacy Carol Davila, 050474 Bucharest, Romania; cristian.hariga@umfcd.ro (C.-S.H.);; 2 Clinic of Plastic Surgery and Reconstructive Microsurgery, Clinical Emergency Hospital of Bucharest, 014461 Bucharest, Romania; 3 Department 1, Discipline Pharmacology, Clinical Pharmacology and Pharmacotherapy, University of Medicine and Pharmacy Carol Davila, 050474 Bucharest, Romania; 4 Ophthalmology Clinic, Central Military Emergency University Hospital ‘Carol Davila’, 010242 Bucharest, Romania

**Keywords:** migraine headache, migraine pathophysiology, migraine trigger sites, pharmacologic therapy, onabotulinumtoxinA injections, surgical decompression

## Abstract

Migraine is a prevalent and disabling neurological disorder with multifactorial origins and complex clinical manifestations. While pharmacologic therapies remain the cornerstone of management, a growing body of evidence highlights the role of extracranial peripheral nerve compression as a significant contributor to migraine pathophysiology in selected patients. This recognition has expanded the therapeutic role of plastic surgery, offering anatomically targeted interventions that complement or surpass traditional medical approaches for refractory cases. From a plastic surgeon’s perspective, optimal migraine care begins with accurate identification of clinical patterns, trigger-site mapping, and the judicious use of diagnostic tools such as nerve blocks and botulinum toxin. Surgical decompression techniques, including endoscopic and open approaches, address compression of the supraorbital, supratrochlear, zygomaticotemporal, greater and lesser occipital, auriculotemporal, and intranasal contact-point trigger sites. Adjunctive strategies such as autologous fat grafting further enhance outcomes by providing neuroprotective cushioning and modulating local inflammation through adipose-derived stem cell activity. Recent advances, including neuromodulation technologies, next-generation biologics, and innovations in surgical visualization, underscore the ongoing shift toward precision-based, mechanism-driven therapy. As understanding of migraine heterogeneity deepens, the integration of surgical expertise with modern neuroscience offers a comprehensive and personalized therapeutic framework. Plastic surgeons, equipped with detailed knowledge of peripheral nerve anatomy and minimally invasive techniques, play an increasingly pivotal role in the multidisciplinary management of refractory migraine.

## 1. Introduction

Migraine is a complex neurological disorder primarily marked by recurrent headaches of moderate to severe intensity, with a prevalence estimated at 14–17%, depending on the source and global region and affecting over a billion people globally. It typically begins in childhood or around puberty and is significantly more common in women, who are affected about three times more often than men. The diagnosis is based on the official ICHD-3 criteria, including pain type, duration, influence on daily life, and associated symptoms [1,2,3,4,5].

Migraine is one of the leading causes of disability worldwide, particularly among young and middle-aged adults. Its disabling nature extends far beyond just the pain of a headache—it affects work, school, family life, and mental health [6].

Migraines have affected humanity for thousands of years, with historical records indicating their presence in ancient civilizations. Mesopotamian texts from around 4000 BC described severe headaches linked to gods and evil spirits, and skull findings suggest trepanation was used to relieve headache pain. Ancient Egyptian and Greek physicians, including Hippocrates, documented migraine symptoms scientifically, breaking away from superstition. Hippocrates described visual disturbances preceding head pain, a hallmark of migraine aura. In the 17th century, Thomas Willis introduced the vascular theory of migraine, suggesting blood vessel dilation as a cause. Later, Edward Liveing and Moebius expanded on neurological and vascular components, emphasizing the role of both brain and blood vessel dysfunctions in migraine development. These historical insights laid the groundwork for the modern understanding of migraine pathophysiology [7,8,9].

Currently, migraine continues to represent a prevalent and disabling neurological disorder with diverse and intricate underlying mechanisms. While pharmacologic therapy remains the foundation of treatment, increasing evidence supports the contribution of extracranial peripheral nerve compression to migraine pathophysiology in selected patients. Despite this, surgical and anatomically targeted interventions remain inconsistently represented in the migraine literature and guidance regarding patient selection, diagnostic pathways and operative strategy is often fragmented or insufficiently defined. Effective management of migraine requires a multidisciplinary team approach including neurologists, pain specialists, plastic and reconstructive surgeons, ophthalmologist and allied health professionals, each contributing with complementary expertise in pharmacologic management, trigger-site identification, interventional therapies and surgical treatment to achieve personalized, mechanism-driven care.

This narrative review aims to bridge this gap by synthesizing current evidence from a plastic surgery perspective, emphasizing trigger-site identification, diagnostic modalities, and surgical decompression techniques, and establishing an integrative overview of the existing therapeutic options and promising future technologies. Plastic surgeons are uniquely positioned to address this mechanism due to their advanced understanding of peripheral nerve anatomy and soft-tissue dynamics, surgical decompression techniques and autologous fat grafting procedures. Beyond operative intervention, in close collaboration with the neurologists, plastic surgeons contribute to accurate trigger-site identification, patient selection and the integration of diagnostic tools such as nerve blocks and onabotulinumtoxinA injections. This paper may serve as a diagnostic and therapeutic framework for clinicians involved in the multidisciplinary management of migraines, including plastic surgeons, neurologists, pain specialists and may also provide adequate guidance for general practitioners involved in migraine headache treatment to optimize patient referral.

## 2. Objectives

Considering the scope and complexity of this scientific domain, this review defines specific objectives intended to structure a comprehensive and progressive analysis of the field, thereby supporting a clear, detailed, and methodologically coherent understanding for the reader, as depicted in Table 1.

## 3. Methodology

This study was designed as a narrative review aimed at synthesizing and critically evaluating the current literature on migraine headache, with a specific focus on migraine pathophysiology, trigger-site identification, and both pharmacologic and surgical treatment strategies. Particular attention was given to the role of extracranial peripheral nerve involvement and the contribution of plastic surgery-based interventions in the management of migraines. A comprehensive literature search was conducted using major academic databases, including Google Scholar, Web of Science, and PubMed. The search strategy incorporated the following keywords and their relevant combinations: migraine headache, migraine pathophysiology, migraine trigger sites, pharmacologic therapy, onabotulinumtoxinA injections, and surgical decompression. Articles were initially screened based on title and abstract, followed by full-text review of the relevant literature. Priority was assigned to original research articles, systematic reviews, and well-designed clinical studies that addressed diagnostic approaches, medical management, and surgical decompression techniques for migraine, ensuring that the selected material provided relevant insights and data. More than this, we included recent studies published up to the year 2025. The selected literature was synthesized descriptively to provide an integrated, mechanism-oriented overview of contemporary migraine management from a plastic surgery perspective.

## 4. Mechanisms and Pathophysiology of Migraines

### 4.1. Triggers for the Migraine

While its exact underlying mechanisms have not been fully clarified, several theories have been proposed to account for both the headache and aura symptoms. The complexity and limited understanding of its development have led researchers to explore a range of contributing biological and social factors, such as hormonal fluctuations, genetic and epigenetic influences, as well as associations with cardiovascular, neurological, and autoimmune disorders [9,10,11].

Genetic factors play a significant role in predisposing individuals to frequent attacks by sensitizing brainstem pain pathways and lowering the threshold for migraine onset. Environmental triggers such as stress, hormonal changes, weather, sleep disturbances, and alcohol further influence attack frequency. Research indicates that migraine has a strong genetic component, though it is unlikely to follow a simple inheritance pattern. Studies suggest a multifactorial mode of inheritance, with both genetic and non-shared environmental factors contributing to migraine susceptibility [9]. Genome-wide association studies (GWAS) have clearly demonstrated the polygenic nature of migraine, identifying over 40 susceptibility genetic loci on different chromosomes linked to migraine without aura [12,13,14].

In addition, a study conducted by Gormley et al. showed that individuals from migraine-affected families showed a higher burden of common genetic variants associated with migraine, as measured by polygenic risk scores (PRS). The effect was observed across all migraine subtypes, including rare forms, with the lowest genetic risk being seen in migraine without aura, while a higher risk was found in migraine with aura and hemiplegic migraine [15]. A more pronounced family history of migraine was likewise linked to an earlier age of onset, increased use of medication, and a higher likelihood of experiencing migraine with aura [16].

The differences between GWASs that have identified stronger links between genetic loci and migraine without aura and other epidemiological studies showing that familial clustering is most pronounced in cases of migraine with aura subtype led to the hypothesis that migraine with aura may be influenced by medium to high-risk genetic variants that are rarer and therefore not well captured by GWAS methodologies [17].

Familial Hemiplegic Migraine (FHM) is a rare, inherited form of migraine characterized by at least some degree of hemiparesis during the aura phase. Unlike common migraines, FHM follows a monogenic inheritance pattern, meaning it is caused by mutations in specific single genes. Several genes have been identified in association with FHM, including CACNA1A, ATP1A2, and SCN1A, all of which play roles in ion transport and neuronal excitability. Despite its rarity, FHM shares many clinical features with common multifactorial migraine, suggesting shared biological pathways in migraine development [18].

Since migraines occur more frequently in females, various hormones have been investigated for their potential role in migraine development. Among them, estrogen has been consistently linked to migraine pathogenesis in numerous studies. Estrogen receptors ERα, ERβ, and G protein-coupled estrogen receptor (GPER) are found in key migraine-related areas such as the trigeminal ganglion, trigeminal nucleus caudalis, and the hypothalamus. While some evidence suggests that estrogen receptor activation may increase the sensitivity of trigeminovascular neurons, the current data supporting this idea remains limited. The concept of estrogen withdrawal as a trigger for menstrual migraine was first proposed by Sommerville in 1972, leading to experimental studies regarding the effect of premenstrual estradiol supplementation in women. However, existing studies exploring this theory were conducted on small sample sizes and had inconsistent definitions, making it difficult to draw firm conclusions, and more robust research is needed using larger, better-defined cohorts to clarify whether estradiol therapy could be an effective migraine treatment [19,20,21].

Among the most probable contributors to migraine headaches are stress, reported in approximately 80% of cases, and hormonal fluctuations, particularly during menstruation, ovulation, or pregnancy, which affect around 65% of individuals with migraine. Skipping meals and weather changes are also commonly implicated, each affecting more than half of patients. Other probable triggers include exposure to bright lights (38%) and alcohol consumption, especially wine. Certain factors are considered possible but less consistent, such as irregular sleep patterns, whether excessive or insufficient, affecting about 50% of sufferers, and aspartame, a food additive. Additional reported triggers include strong odors (40%), neck pain (38%), smoking (36%, though unproven), late sleeping habits (32%), heat exposure (30%), specific foods like chocolate and tyramine-rich products (27%), exercise (22%), and even sexual activity, though the latter is noted in only 5% of cases [22,23].

### 4.2. Peripheral Trigger Points

Increasing evidence implicates that extracranial peripheral nerves (supraorbital, supratrochlear, zygomaticotemporal, auriculotemporal, and greater and lesser occipital nerves) act as “trigger points” susceptible to compression by adjacent musculature, fascia, or vascular loops. Persistent nociceptive input can amplify central processes and perpetuate migraine cycles [24].

**Figure 1:**
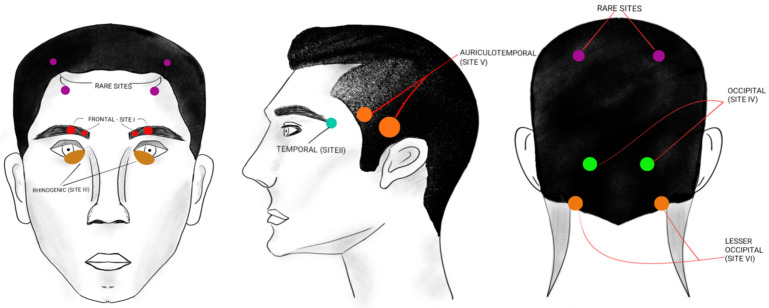
Anatomy of migraine trigger points: Site I—supraorbital and supratrochlear nerve, Site II—zygomaticotemporal nerve, Site III—nasal branches of the trigeminal nerve, Site IV—greater occipital or the lesser or third occipital nerves, Site V—auriculotemporal nerve and Site VI—lesser occipital nerve [25].

The location and pattern of headache onset in migraine patients can assist in identifying the most probable anatomical trigger points, as summarized in Table 2 and depicted in Figure 1.

Identifying the trigger point for migraine headaches is very important for planning the individualized treatment of the patient.

### 4.3. Pathophysiology

The vascular hypothesis of migraine was one of the earliest explanations for the mechanisms underlying migraine attacks. According to this theory, the narrowing of cerebral blood vessels, or vasoconstriction, leads to the aura phase experienced by some individuals before the onset of headache. This is then followed by vasodilation, or the widening of these vessels, which is thought to cause the characteristic throbbing headache associated with migraines. Support for this idea came from clinical observations. For example, vasodilator substances such as nitroglycerin were known to trigger migraine-like headaches, while vasoconstrictive drugs like ergotamine and triptans were effective in relieving migraine symptoms. These findings reinforced the belief that the dilation of blood vessels played a central role in the generation of migraine pain. However, while the vascular hypothesis contributed significantly to early understanding and treatment approaches, it is now considered incomplete. Advances in neuroscience have revealed that migraines are more complex and cannot be fully explained by vascular changes alone. The current understanding recognizes migraines as neurovascular disorders, involving not just blood vessels but also the nervous system [27,28,29].

Although the brain itself lacks pain sensitivity, a rich plexus of nociceptive nerve fibres that originate in the trigeminal ganglion supply the pial, arachnoid, and dural blood vessels, including major vessels such as the superior sagittal sinus, middle meningeal artery, and large cerebral arteries. When these pain-sensitive areas, especially the dura mater, are activated through mechanical, chemical, or electrical means, they can trigger headache pain that closely resembles a migraine attack and are often associated with other symptoms such as photophobia and nausea [30,31].

The trigeminovascular system serves as the anatomical and physiological foundation of the characteristic throbbing pain of migraine headache. It comprises peripheral sensory axons (craniofacial nociceptive afferents) that innervate the meninges and intracranial blood vessels, as well as central projections that converge in the trigeminocervical complex, the region that serves as the primary central relay point in the craniofacial pain pathway. Craniofacial nociceptive afferents are thinly myelinated A-delta fibers or unmyelinated C fibers that originate from cell bodies located in the trigeminal ganglion and the dorsal root ganglia of the C1–C3 cervical spinal nerves. Their central projections terminate in the trigeminocervical complex, which includes the trigeminal subnucleus caudalis and the dorsal horn of the upper cervical spinal cord. Craniofacial nociceptive afferents often express neuropeptides such as calcitonin gene-related peptide (CGRP) and substance P. The activation of the trigeminovascular pain pathway leads to the release of neuropeptides like calcitonin gene-related peptide (CGRP) and pituitary adenylate cyclase-activating polypeptide (PACAP) at the dura mater, contributing to vasodilation and pain sensitization [10,29]. Sensory signals from the trigeminocervical complex ascend to various brainstem, thalamic, hypothalamic, and basal ganglia nuclei, which project further to a wide range of cortical regions—including the somatosensory, insular, motor, parietal association, retrosplenial, auditory, visual, and olfactory cortices. These cortical areas process the emotional, cognitive, and sensory aspects of pain, contributing to hallmark migraine symptoms such as photophobia, phonophobia, cognitive disturbances, osmophobia, and allodynia [1,32,33].

Researchers aiming to understand how hypothalamic and brainstem neurons trigger migraine attacks—especially during the prodromal phase—have proposed two main theories that offer insight into the neurological underpinnings of migraine. The first theory suggests that hypothalamic neurons, which regulate emotional and physiological balance, may initiate migraine by shifting the autonomic tone toward parasympathetic dominance in the meninges. This shift can activate meningeal nociceptors through the release of neurotransmitters such as acetylcholine, nitric oxide, and vasoactive intestinal peptide (VIP) from parasympathetic fibers of the sphenopalatine ganglion. These substances contribute to vasodilation, plasma protein extravasation, and local inflammation, all of which can lead to the activation of pain pathways. Evidence supporting this mechanism includes the dense parasympathetic innervation of meningeal blood vessels, the ability of parasympathetic output to influence trigeminovascular pain transmission, and clinical observations that blocking the sphenopalatine ganglion can relieve migraine pain [1,34,35].

The second theory focuses on central mechanisms, proposing that hypothalamic and brainstem neurons lower the threshold for nociceptive signal transmission from the thalamus to the cortex. These brain regions send neuromodulatory input—via neurotransmitters such as dopamine, serotonin, histamine, and orexin—to thalamic relay neurons involved in pain processing. Depending on the specific neurochemical environment, these inputs can either increase or decrease the excitability of thalamic neurons, thereby influencing whether a migraine attack is triggered. This theory explains the variability of migraine onset in response to internal and external stimuli such as stress, hunger, lack of sleep, or sensory inputs. It also introduces the concept of “allostatic load”, the brain’s burden in managing physiological and emotional stress, as a possible factor determining whether a prodrome leads to a full migraine [1,36,37].

Cortical spreading depression (CSD), first described by Leão, is believed to underlie the phenomenon of migraine aura. It involves a slow-moving wave of intense electrical activity, often referred to as a “brain tsunami,” which is followed by a prolonged phase of suppressed neural activity and reduced blood flow, which defines the depression phase of CSD. Cortical spreading depression causes widespread changes in the function of neurons, glial cells, and blood vessels. After this initial surge in activity, neurons enter a state of prolonged hyperpolarisation, where their electrical balance is reversed, making it difficult for them to send signals. During this time, both spontaneous brain activity and responses to external stimuli are significantly suppressed. While spontaneous activity may start to recover within 5 to 10 min, full recovery can take up to an hour. In contrast, evoked activity, meaning brain responses to external inputs, can take 15 to 30 min to return to normal [38,39]. CSD also triggers an inflammatory response in the brain. It activates pannexin-1 megachannels, which lead to the activation of caspase-1 and the release of pro-inflammatory molecules. These molecules stimulate the nuclear factor kappa-B (NF-κB) pathway in astrocytes, thereby relaying inflammatory signals that activate trigeminovascular nociception. The sensory and visual symptoms of migraine aura closely mirror the timing and progression of CSD [40]. Positive symptoms, such as flickering lights or zig-zag patterns, are believed to be caused by the advancing wave of neural excitation, while negative symptoms, like blind spots or vision loss, are linked to the subsequent phase of neural suppression. This strong similarity between migraine aura and cortical spreading depolarisation helps explain the neurological patterns experienced during an aura [38].

## 5. Clinical Presentation

### 5.1. Classification

**Figure 2:**
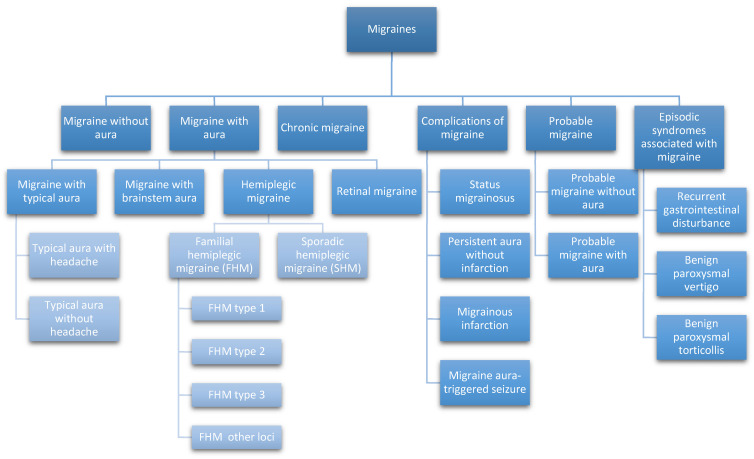
ICHD-3 classification of migraines [41].

The classification of migraines, according to the 3rd edition of the International Classification of Headache Disorders (ICHD-3) developed by the International Headache Society (IHS) is depicted in Figure 2.

Migraines without aura are the most common form of migraine, in both children and adults, and are characterized by recurrent headache attacks that occur without preceding neurological symptoms. Attacks typically have a polyphasic course, consisting of a prodrome, headache phase, and postdrome. These headaches are typically moderate to severe in intensity, often throbbing or pulsating, and usually unilateral. They may last from 4 to 72 h and are commonly aggravated by physical activity. Associated symptoms often include nausea, vomiting, photophobia and phonophobia, and pain aggravated by movement. The overall presentation is diverse and variable among individuals [4,42].

Migraine aura is a temporary neurological symptom that often precedes or occurs with a migraine headache due to sudden changes in brain activity. Approximately 25–30% of individuals with migraines experience aura symptoms, most commonly visual auras (over 90% of cases), which may be accompanied by neurological signs such as dizziness, numbness, memory impairment, or aphasia. Aura symptoms consist of visual and/or sensory and/or speech/language symptoms, but no motor weakness, that develop gradually over about 5–20 min and last for less than 60 min, with complete reversibility. The typical visual manifestations of migraine aura include positive symptoms, such as flashing lights, scintillating scotomas, visual distortions, and heat wave–like sensations, and negative symptoms, including dark spots, blurred vision, or homonymous hemianopsia. Other eye-related or neurological conditions can mimic migraine aura, but a defining feature of migraine aura is its gradual onset and progression of positive visual symptoms [41,43,44,45,46].

Typical aura without headache, also called acephalgic migraine, migraine equivalent, or migraine accompaniment, is a migraine aura that occurs without any associated headache simultaneously or within 60 min. It is seen in about 4% of migraine patients and may occur at some point in up to 38% of those with migraine headaches. It is more common in women (3%) than in men (1%) and tends to occur later in life, especially among older adults. Some studies suggest a bimodal age distribution, with peaks between ages 20–39 and 60–69. A diagnosis of migraine aura without headache should be made only after ruling out transient ischemic attacks and seizure disorders [41,47,48].

Migraine with brainstem aura (previously called basilar-type migraine) is a rare subtype of migraine with aura that primarily affects children, adolescents, and young adults, and it is characterized by neurological symptoms originating from the brainstem or both cerebral hemispheres simultaneously, but without motor weakness. For diagnosis, Yamani et al. suggested the existence of at least three of the following fully reversible brainstem symptoms: dysarthria, vertigo, tinnitus, hypoacusis, diplopia, ataxia not attributable to sensory deficit or weakness, decreased level of consciousness (GCS ≤ 13), and simultaneously bilateral visual symptoms and/or simultaneously bilateral paresthesias, but without motor or retinal symptoms [46,47,49]. Because the current diagnostic criteria lack specificity, a thorough face-to-face evaluation by a neurologist (including a detailed history, neurological examination, and neuroimaging such as MRI, along with other relevant tests to exclude alternative diagnoses) is essential to achieve an accurate and reliable diagnosis [50].

Hemiplegic migraine is a severe and rare (prevalence ~0.01%) subtype of migraine with aura, characterized by transient motor weakness (hemiparesis) in addition to typical aura symptoms such as visual, sensory, or speech disturbances. It can be familial (FHM) or sporadic. Familial HM is diagnosed when at least one first- or second-degree relative experiences similar attacks. Diagnosis relies on the detailed patient history, neurological examination during attacks, and exclusion of other conditions like epilepsy, stroke, encephalitis, and secondary headache syndromes. Three genes (CACNA1A, ATP1A2, and SCN1A) are associated with familial HM, with mutations facilitating cortical spreading depolarization. Genetic testing can confirm the diagnosis, but a negative result does not exclude HM. Patients without mutations typically have a milder phenotype similar to common migraine. Additional tests, such as brain imaging, EEG, and cerebrospinal fluid analysis, are primarily used to rule out other causes of focal neurological symptoms [51,52,53].

Retinal migraine is characterized by transient, fully reversible monocular visual disturbances, including scotomas, visual dimming, blurred vision, and occasionally complete unilateral vision loss. These episodes typically last between 5 and 20 min, with a maximum duration of 60 min, and may be accompanied or followed by a unilateral migraine headache. Although often benign and self-limited, retinal migraine is clinically significant due to its potential association with permanent visual complications. Epidemiologically, it is far less common than other migraine subtypes, with onset typically in the second to fourth decades of life and frequent association with personal or family history of migraine. Well-established precipitants mirror those of migraine with aura and include emotional stress, hypertension, hormonal contraceptives, dehydration, high-altitude exposure, hypoglycemia, smoking, and excessive heat [54,55,56,57]. The pathophysiology of retinal migraine remains incompletely understood. Proposed mechanisms include transient vasospasm of the retinal or ciliary circulation and retinal neuronal spreading depression analogous to cortical spreading depression observed in migraine aura. During attacks, fundoscopic examination or fluorescein angiography may demonstrate retinal hypoperfusion, segmental vasoconstriction, or delayed filling of the central retinal artery or its branches; however, interictal examinations are frequently normal. Despite its generally benign nature, retinal migraine has been associated with serious complications, including retinal artery or vein occlusions, choroidal or optic nerve ischemia, retinal infarction, and permanent vision loss [54,58,59,60]. Diagnosis is clinical and requires strict adherence to migraine-with-aura criteria as defined by the International Classification of Headache Disorders, including: fully reversible monocular visual phenomena confirmed by visual field testing or patient drawings, gradual spread of symptoms over at least 5 min, duration of 5–60 min, and a migraine headache occurring within 60 min of the visual disturbances. Because retinal migraine is a diagnosis of exclusion, clinicians must first differentiate monocular from binocular symptoms and systematically rule out other causes of transient monocular visual loss, including amaurosis fugax from carotid or cardiac emboli, giant cell arteritis, optic neuritis, increased intracranial pressure, orbital apex lesions, and anterior ischemic optic neuropathy. Ancillary testing, such as carotid ultrasonography, echocardiography, inflammatory markers, and fluorescein angiography, may be required when the clinical picture is atypical or vascular pathology is suspected [3,56,60,61].

Chronic migraine, as classified by the International Headache Society, is defined as headache occurring on 15 or more days per month for over three months, with at least eight of those days showing features of migraine or responding to migraine-specific treatments like triptans or ergotamines. This condition affects approximately 2–4% of the population and is associated with significantly reduced quality of life and a higher incidence of comorbidities compared to episodic migraine. Chronic migraine often develops from episodic migraine, a progression sometimes referred to as “transformed migraine.” A major contributing factor to this transformation is the frequent use of acute headache medications, such as opioids, triptans, ergotamines, or combination analgesics on more than nine days per month, or NSAIDs, aspirin, or acetaminophen on more than 14 days. Importantly, about half of the individuals initially diagnosed with chronic migraine may revert to episodic migraine once the overused medications are withdrawn. Other risk factors for progression to chronic migraines are female gender, low socioeconomic status, obesity, baseline headache frequency, stressful events, comorbid pain and other comorbidities such as depression and anxiety [62,63,64].

### 5.2. Clinical Phases of Migraines

**Figure 3:**
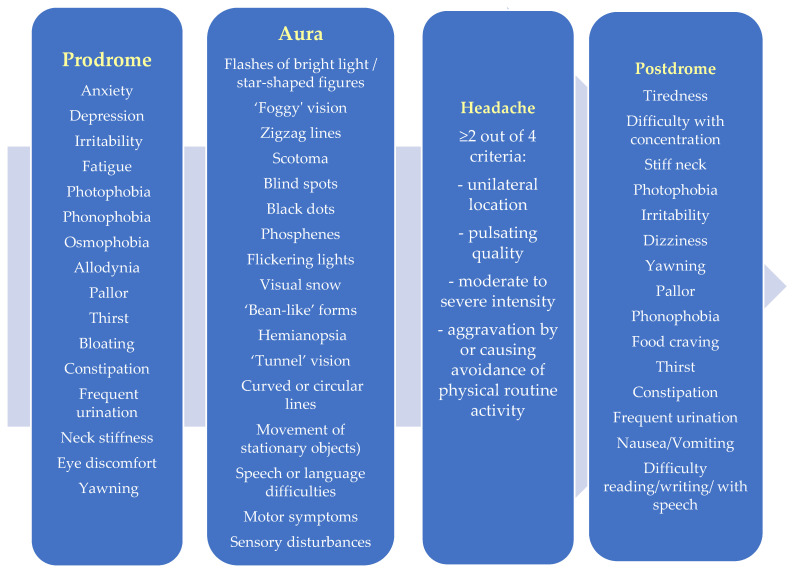
The four phases of migraines [3,43,66,67,68,69,70,71,72].

The migraine attack is a multiphasic event that typically unfolds in four stages: premonitory (prodrome), aura, headache, and postdrome, as shown in Figure 3. Each phase presents a complex and changing array of symptoms, and the entire process can last up to a week, significantly severely affecting a patient’s daily life [65].

The prodrome phase has predictive value, offering a therapeutic window for early intervention in order to reduce the impact of migraines on quality of life. One-third of migraine patients experience prodromal symptoms regardless of their migraine type. The prodrome phase consists of symptoms preceding a migraine attack by 2–48 h, occurring before the aura in migraine with aura and before the onset of headache in migraine without aura, that can last from hours to days and often cause varying degrees of discomfort [3,66]. Prodromal symptoms of migraine are generally grouped into four categories. Neuropsychiatric symptoms include anxiety, depression, irritability, mood change, asthenia, somnolence, and concentration difficulties. Sensory symptoms involve heightened sensitivity,6 such as photophobia (light sensitivity), phonophobia (sound sensitivity), osmophobia (sensitivity to smells), and allodynia (pain from normally non-painful stimuli). Autonomic symptoms affect bodily functions and can include bloating, nausea, pallor, constipation, frequent urination, and increased thirst. Finally, general symptoms encompass yawning, neck stiffness, and eye discomfort. The prodrome phase serves as an important early indicator of an impending migraine attack [66,67,68].

The aura phase represents a transient neurological symptom that typically occurs before or during the headache phase. It usually develops gradually over several minutes and lasts less than an hour, being completely reversible. Auras most commonly involve visual disturbances (98–99% of migraines with aura), such as flashing lights, zigzag patterns, blind spots, or tunnel vision. However, they can also affect other neurological functions, causing sensory changes like tingling or numbness (36% of cases), speech or language difficulties (10% of cases), motor weakness, or brainstem-related symptoms [43,69].

The headache phase lasts between 4 and 72 h if left untreated or if treatment is ineffective. It is characterized by a combination of symptoms, including pain that is usually located on one side of the head, although bilateral pain is not uncommon (40% of patients experience pain on both sides of the head during attacks). Pain is often described as pulsating or throbbing in nature. The intensity of the headache is moderate to severe, enough to interfere with daily activities, and it tends to worsen or be triggered by routine physical movements such as walking or climbing stairs, causing the person to avoid these activities. These features help distinguish migraines from other types of headaches. In addition to the characteristic headache, migraines are frequently accompanied by other symptoms such as sensitivity to light (photophobia), sensitivity to sound (phonophobia), nausea, and vomiting. These associated symptoms contribute to the overall disabling nature of migraine attacks [3,70,71].

The postdrome phase is the period after the throbbing headache resolves but before the patient feels fully recovered, and usually lasts between 18 and 25 h. During this phase, many migraine sufferers experience various non-headache symptoms that can significantly affect their ability to return to normal function. Studies show that about 81% of migraine patients report at least one such symptom, which can include neuropsychiatric, sensory, gastrointestinal, and general systemic issues. Common symptoms are tiredness, difficulty concentrating, and neck stiffness [72].

## 6. Diagnosis

The diagnosis should be made by a neurologist. Migraine diagnosis relies primarily on a thorough medical history, supported when needed by diagnostic tools that help apply the ICHD-3 criteria. Physical examination usually serves to support the diagnosis, while tests such as imaging, blood work, or lumbar puncture are reserved for cases in which a secondary headache cause is suspected. A complete history should document when headaches began, how long and how often they occur, the nature and severity of the pain, triggers or relievers, associated symptoms such as light or sound sensitivity or nausea, any aura features, and details of current or past headache treatments. These elements are required to properly use the ICHD-3 framework.

Differential diagnoses for migraine include other primary headache disorders such as tension-type headache, cluster headache, or secondary headaches associated with conditions like subarachnoid haemorrhage, subdural hematoma, transient ischaemic attack, stroke, epilepsy, arteriovenous malformations, intracranial tumours, meningitis, or intracranial hypertension or hypotension [70,73].

Routine neuroimaging is not needed for patients whose headaches fit the typical pattern of migraine, who have a normal neurological exam, and who show no atypical features or red flags. Imaging can be considered in patients with presumed migraine when specific worrisome features are present. These include an atypical, prolonged, or persistent aura; a notable rise in headache frequency or severity; any change in the usual headache pattern; a first or worst migraine episode; migraine with brainstem symptoms; episodes of confusion; motor deficits such as those found in hemiplegic migraine; late-onset aura-related symptoms; aura without accompanying headache; consistently one-sided (side-locked) pain; or headaches that develop after head trauma. Patients who present with worrisome symptoms or abnormal examination findings are more likely to have clinically significant abnormalities and should undergo imaging. In addition, “red flag” features—such as fever, immunosuppression, papilledema, or pregnancy—especially when occurring together, increase the likelihood of a secondary cause and justify further evaluation [74,75].

## 7. Treatment

### 7.1. Pharmacologic Treatment

Pharmacological treatment is the main therapeutic strategy for patients with migraine and includes two categories of antimigraine drugs: those for stopping acute attacks (symptomatic/abortive medications) and those used prophylactically [28,76]. Abortive medications are most effective when administered as early as possible after symptom onset [77]. In some migraine patients presenting with digestive symptoms, such as nausea, vomiting and gastric stasis, the bioavailability of oral medications is reduced; in these cases, alternative non-oral formulations (suppositories, nasal sprays, injectable solutions) should be considered [78]. A potential adverse effect of frequent use of acute medications is the development of medication-overuse headache (MOH), a condition where headache frequency and intensity increase with overuse of abortive drugs [79]. Abortive medications may act nonspecifically (analgesics, NSAIDs, glucocorticoids, antiemetics) or specifically (ergotamines, triptans, ditans, CGRP antagonists) [80].

- Analgesics—Paracetamol (acetaminophen)—is the safest but least effective abortive agent [81]. The usual dose for attack termination is 1 g. Caution is advised in patients with liver disease, where the maximum daily dose should not exceed 2.5–3 g [81,82,83].
- NSAIDs are the most commonly used agents, often self-medicated. Ibuprofen, with a half-life of about 2 h and rapid onset, is administered in doses of 800–1200 mg per attack [84]. Soft-gel capsules allow faster absorption and a faster action onset [85]. Naproxen (500 mg per attack) has a prolonged duration of action (~14 h), but slower onset (t_max_ ~ 2 h) [84]. Diclofenac is likely the most effective NSAID for migraine, with rapid peak plasma levels; usual dose per attack is 50 mg [84,86]. Acetylsalicylic acid used in doses of 500 to 1000 mg has a rapid onset (t_max_ ~ half an hour) [84]. Other potent NSAIDs include indomethacin (25–100 mg per attack) and ketorolac (30–60 mg per attack) [84,87]. Available formulations include oral (capsules, tablets, powders, syrups), suppositories (Indomethacin, Diclofenac), injectable solutions (Ketorolac, Diclofenac), and nasal spray (Ketorolac) [88,89]. Their drawbacks include nephrotoxicity and gastrointestinal side effects [90]. To prevent MOH, usage should be limited to 15–20 days per month [79].
- Triptans are considered the gold standard in antimigraine therapy [91]. They are selective agonists of 5-HT_1B_ and 5-HT_1D_ receptors and the most effective agents for aborting migraine attacks [92]. Sumatriptan is the prototype triptan. Based on duration of action, triptans are classified as short-acting (Sumatriptan, Rizatriptan, Zolmitriptan, Eletriptan), intermediate (Naratriptan), and long-acting (Frovatriptan) [91]. Sumatriptan is available as tablets, nasal spray, auto-injector pen, and nasal powder [93]. Side effects include pressure sensations in throat and chest, flushing, warmth, and blood pressure increase. It is contraindicated in patients with cerebrovascular disease, chronic coronary artery disease, peripheral artery disease, uncontrolled hypertension, and severe liver disease. To prevent MOH, usage should not exceed 10 days per month [94].
- Ditans act by selectively blocking 5-HT1F receptors, thereby reducing neurogenic inflammation. Lasmiditan was approved by the FDA in 2019 for the acute treatment of migraine attacks, with or without aura [95].
- Caffeine is frequently combined with analgesics [96]; it is a methylxanthine with vasoconstrictive effects via phosphodiesterase inhibition, increasing circulating catecholamines, and adenosine receptor antagonism [97]. Clinical guidelines recognize the efficacy of analgesic + caffeine combinations. Common formulations include paracetamol + caffeine and a triple combination of paracetamol + aspirin + caffeine. In the US, combinations with butalbital, NSAIDs, and caffeine exist but carry risks of dependence, sedation, and MOH [98].
- CGRP antagonists (“gepants”) are small lipophilic molecules used in acute migraine treatment [99]. CGRP is a potent vasodilator and neurogenic inflammation mediator in migraine pathogenesis [100]. Blocking CGRP or its receptor reduces vasodilation and inflammation, decreasing migraine intensity and frequency [101]. Gepants do not cause vasoconstriction and are safe in patients with high cardiovascular risk [102]. Approved agents include Rimegepant (oral, EMA and FDA approved for acute and preventive treatment), Ubrogepant (oral, FDA approved for acute treatment), and Zavegepant (oral and nasal, FDA approved for acute treatment) [103]. They do not cause dependence or MOH and are good options for patients at risk for MOH [104].
- Ergot derivatives (Ergotamine and Dihydroergotamine) are available only in the US with restrictions [105]. The EMA withdrew authorization for these drugs in Europe due to risks of multiorgan fibrosis and ergotism (severe vasoconstriction and neurotoxicity) [106]. Dihydroergotamine (DHE 45) is available as nasal spray and injectable formulation, useful in status migrainosus [107,108]. Side effects include nausea, muscle cramps, and chest constriction sensations [105].
- Isometheptene derivatives are older agents with strong vasoconstrictive effects via direct α1-adrenergic agonism and increasing synaptic norepinephrine [109]. Used in combination with paracetamol and/or caffeine, they have been restricted in USA [110].

Other abortive classes include:

- Dopamine antagonist antiemetics like Prochlorperazine and Metoclopramide, effective as adjunctives, often administered intravenously in emergency settings [111]
- Glucocorticoids used as adjuncts in severe or refractory migraines reduce neurogenic inflammation and prevent rapid recurrence, useful in status migrainosus [112]
- Intravenous sodium valproate in refractory cases rapidly reduces pain severity [113]
- Intravenous magnesium sulfate, used off-label in emergencies, reduces neuronal excitability and regulates vascular tone [114]
- Opioids, last-resort options for severe refractory migraine, carry significant risks and are generally contraindicated [115]

Migraine prophylaxis aims to reduce the number, duration, and severity of attacks by at least 50% after a minimum of 3 months of treatment [116]. Although preventive therapy is underused [117], a lot of options are available:

- Beta-blockers (Propranolol, Nadolol, Timolol) reduce neuronal excitability and induce vasoconstriction via β2 antagonism. Contraindicated in asthma, peripheral artery disease, AV block, bradycardia, and hypotension [118]
- Antiepileptics (Topiramate, Valproate, Gabapentin) stabilize neuronal membranes and reduce cortical excitability by modulating ion channels and GABAergic transmission [119]
- Tricyclic antidepressants and SNRIs (Amitriptyline, Nortriptyline, Venlafaxine) modulate central serotonergic and noradrenergic pathways and are useful for psychiatric comorbidities [120]
- Calcium channel blockers (Flunarizine, Verapamil) reduce calcium influx in smooth muscle and neurons, preventing abnormal vasodilation [119]. Flunarizine causes weight gain and sedation [121]
- Sartans (Candesartan, Telmisartan) block AT_1_ receptors, reducing vasoconstriction and neurogenic inflammation, effective especially in hypertensive patients [122]
- Monoclonal antibodies against CGRP or its receptor (Erenumab, Galcanezumab, Fremanezumab, Eptinezumab) administered monthly or quarterly [123]
- CGRP antagonists were the first oral agents specifically designed to prevent migraines. Gepants of the second generation, like Rimegepant and Atogepant, are indicated as preventive treatment of episodic migraines in adult patients [124,125]. Croop et al., in a phase 2/3, randomised, double-blind, placebo-controlled trial, demonstrated that oral rimegepant, administered every other day, is effective for the preventive treatment of migraine. The treatment was well tolerated, with a safety profile comparable to placebo, supporting its role as a preventive CGRP receptor antagonist in migraine management [126].
- Other therapies: medicinal plants (butterbur, feverfew, ginkgo, marjoram) with variable evidence [127], oral magnesium supplementation in deficient patients [128], vitamin B2 (riboflavin 400 mg/day) [129], vitamin D [130], combinations of B6, B12 and folic acid to reduce homocysteine [131].

### 7.2. OnabotulinumtoxinA Injections

OnabotulinumtoxinA (botulinum toxin type A) has emerged as an established prophylactic therapy for chronic migraine. Its effect on migraine is not yet fully understood, but it is likely multifactorial. Its actions may involve muscle fibers, autonomic fibers, and possibly pain fibers. BoNT-A appears to reduce peripheral sensitization and may also influence the central processing of pain, either directly or indirectly [132].

Several mechanisms have been proposed to explain its action, such as the inhibition of neurochemical and protein exocytosis in both motor and sensory systems, the suppression of proinflammatory cell, neurotransmitter, and excitatory neuropeptide release (substance P, calcitonin gene-related peptide, and glutamate), and the blockade of soluble N-ethylmaleimide-sensitive factor attachment protein receptor activity [133].

The PREEMPT clinical program demonstrated that onabotulinumtoxinA is an effective, safe, and well-tolerated preventive treatment for chronic migraine (CM) in adults. The program included two large phase 3, multicenter clinical trials (PREEMPT 1 and PREEMPT 2) involving 1384 patients. Each study had a 24-week double-blind, placebo-controlled phase, followed by a 32-week open-label phase. All participants received at least 155 units of onabotulinumtoxinA injected into 31 sites across 7 head and neck muscles using a fixed-site, fixed-dose protocol (5 U per injection in 0.1 mL). An additional up to 40 units could be administered to 8 more sites in 3 muscles using a follow-the-pain approach. This brought the total dose to 155–195 units [134,135].

From a procedural standpoint, BoNT-A is typically administered following the standardized 31-site PREEMPT injection protocol, with site locations fixed at the corrugators (5U each side), procerus (5U one side), frontalis (10U each side), temporalis (20U each side), occipitalis (15U each side), cervical paraspinal (10U each side), and trapezius muscles (15U each side). Treatment sessions are performed at 12-week intervals, and clinical benefit often becomes evident after the second session, with continued improvement over subsequent cycles [134]. The protocol is depicted in Table 3 [136].

### 7.3. Sphenopalatine Ganglion Block

The sphenopalatine ganglion (SPG) block has emerged as a treatment option of increasing interest due to its demonstrated efficacy in managing migraine as well as other headache and facial pain syndromes [137,138].

The sphenopalatine ganglion, known also as pterygopalatine ganglion or Meckel’s ganglion, represents a major extracranial parasympathetic relay situated within the pterygopalatine fossa and interconnected with autonomic, sensory and motor pathways [138,139]. Preganglionic parasympathetic fibers arise from the superior salivatory nucleus and course via the nervus intermedius and greater petrosal nerve, while sympathetic fibers from the superior cervical ganglion reach the ganglion through the deep petrosal nerve; together, these fibers form the Vidian nerve before entering the SPG. Sensory contributions are provided by maxillary nerve branches (V2), mainly through orbital, nasal and palatine branches. Parasympathetic fibers synapse within the ganglion, and postganglionic projections supply secretomotor innervation to the nasal, palatal, and pharyngeal mucosa, the lacrimal gland, and perivascular structures of the meninges and cerebral circulation, whereas sympathetic fibers traverse without synapsing [138,140,141]. Because it serves as the principal parasympathetic relay to cranial structures, the SPG is involved in the pathophysiology of migraine and trigeminal autonomic cephalalgias. Activation of the superior salivatory nucleus and the trigemino-autonomic reflex can lead to increased parasympathetic discharge through the SPG, promoting cranial vasodilation, release of vasoactive inflammatory mediators and activation of meningeal nociceptors. These processes contribute to the sensory and autonomic manifestations characteristic of acute migraine attacks. Additionally, modulation of afferent sensory fibers projecting to the trigeminal nucleus caudalis provides a plausible mechanism by which SPG blockade may attenuate central sensitization and influence migraine pain processing [138,139,142].

The SPG intervenes in modulating craniofacial nociception and parasympathetic outflow; therefore, strategies addressing its block represent a promising therapeutic option for headaches with autonomic symptoms or trigeminal involvement [139,140].

Initially described by Sluder in 1908 in the context of “sphenopalatine neuralgia”, SPG interventions have expanded to include numerous modern modalities. However, for migraine management specifically, therapeutic strategies can be divided into non-invasive transnasal topical techniques and image-guided needle-based approaches aiming to access the pterygopalatine fossa [139].

Non-invasive transnasal topical techniques include cotton-tipped applicators, catheter-based devices or sprays instilled transnasally toward the region of the sphenopalatine foramen. Despite being relatively simple procedures with a favourable safety profile, debate persists regarding the extent to which topically applied agents actually reach the PPF and consequently the SPG. A recent cadaveric study reported by Istenič et al. demonstrated that bupivacaine applied on cotton swabs and endoscopically guided placement adjacent to the sphenopalatine foramen can reliably reach the pterygopalatine fossa, supporting the physiological feasibility of a transnasal approach to pterygopalatine ganglion block. The pattern of anaesthetic spread indicates diffusion as the primary transport mechanism, underscoring that clinical effectiveness depends on precise intranasal placement, adequate contact time and the use of sufficiently concentrated formulations. Overall, the findings reinforce that properly executed transnasal topical techniques can deliver local anaesthetic to the target compartment and should be optimized accordingly in clinical practice [143].

Flexible catheters and commercially developed devices are also used for transnasal placement of local anaesthetic to the posterior nasal cavity [139].

Non-invasive approach for SPG block was also described using an FDA-approved device called Tx360^®^ nasal applicator (Tian Medical Inc., Lombard, IL, USA). The Tx360 device is presented as a simple, cost-efficient tool that can be rapidly deployed, including in emergency settings. It enables accurate delivery of local anaesthetic without the use of a needle and is associated with minimal adverse effects [144,145].

A double-blind, randomized, placebo-controlled pilot study reported by Cady et al. investigated the acute and longer-term effects of repetitive transnasal sphenopalatine ganglion blockade with 0.5% bupivacaine delivered via the Tx360^®^ device in chronic migraine. In the acute-phase trial, forty-one adults with well-defined chronic migraine were randomized 2:1 to receive 12 bilateral SPG blocks with bupivacaine or saline over 6 weeks while maintaining stable preventive therapy; bupivacaine produced significantly greater reductions in headache intensity at 15 and 30 min, with sustained benefit at 24 h and improved headache-related impact, with only mild transient adverse effects. Follow-up analysis of the same cohort demonstrated trends toward sustained clinical benefit, including fewer headache days, reduced acute medication use and improvements in multiple quality-of-life domains for up to six months after treatment, whereas changes in the saline group were minimal. Collectively, these findings suggest that repetitive SPG blockade with bupivacaine delivered by the Tx360^®^ device is well tolerated and may offer both meaningful acute relief and potential longer-term benefit in chronic migraine, supporting the need for larger, adequately powered trials [146,147].

Transnasal sphenopalatine ganglion blockade with the Sphenocath^®^ device (Dolor Technologies LLC, Riverton, UT, USA), as described in the study reported by Binfalah et al., is a noninvasive technique in which a flexible, curved catheter is advanced through the nasal passage and positioned above the middle turbinate to deliver 2 mL of 2% lidocaine directly to the mucosa overlying the SPG. The procedure is simple and can be performed in the outpatient setting with minimal preparation. In this cohort of 55 patients with acute migraine, the technique produced rapid and sustained pain relief, with the majority achieving complete headache freedom at 15 min, 2 h and 24 h, and most reporting “good” or “very good” global improvement. Adverse events were mild, transient and limited to expected local effects such as throat numbness, nasal discomfort, dizziness and nausea. The authors concluded that transnasal SPG blockade using Sphenocath^®^ is an effective, fast-acting and well-tolerated option for acute migraine treatment [138].

A series of invasive approaches allows targeted delivery of local anaesthetic into the pterygopalatine fossa and are generally performed under fluoroscopic, CT or ultrasound guidance. These include: infrazygomatic approach (posterior or lateral-infrazygomatic trajectories), suprazygomatic approach, transoral approach via the greater palatine canal and other percutaneous variants depending on anatomical configuration and imaging modality.

Jerman et al. conducted a cadaveric study comparing injectate distribution and safety between ultrasound-guided posterior infrazygomatic and transoral (via greater palatine canal) approaches to the pterygopalatine fossa in 13 fresh human heads. After vascular perfusion, both approaches were performed bilaterally with CT confirmation of needle position, followed by injection of a methylene blue-iodinated contrast mixture and subsequent imaging and anatomical dissection. The posterior infrazygomatic approach typically positioned the needle at the pterygomaxillary fissure and produced a broad spread of injectate to the entire pterygopalatine fossa, consistently staining the maxillary artery and nerve, vidian nerve, sphenopalatine ganglion and lateral pterygoid muscle; however, it was associated with an injury to the maxillary artery and temporal branch of the facial nerve. In contrast, the transoral approach reliably accessed the inferior portion of the fossa via the greater palatine canal, yielding a more inferiorly localized distribution focused around the maxillary artery and greater palatine canal, with a reported bony injury to the lateral pterygoid plate and no major neurovascular damage. The authors conclude that the posterior infrazygomatic approach is preferable when a more global block of pterygopalatine fossa contents (maxillary nerve, SPG, maxillary artery) is desired, whereas the transoral route may be better suited for targeting palatine nerves or the inferior fossa and that needle end-position and approach should be selected according to specific clinical objectives [148].

**Figure 4:**
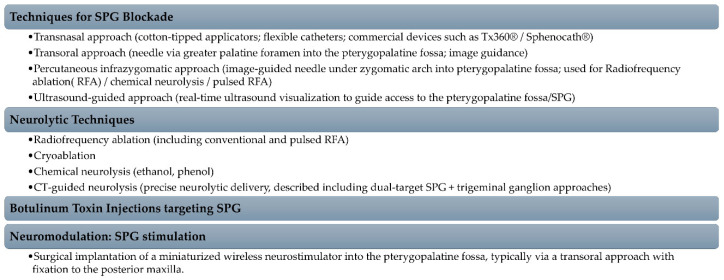
Synthetic presentation of available techniques targeting the sphenopalatine ganglion for migraine treatment [139].

Bautista et al. extensively reviewed the techniques that have been developed to block or modulate the SPG, ranging from non-invasive to percutaneous and surgically implanted modalities, these findings being synthesized by Figure 4 [139].

### 7.4. Surgical Decompression

#### 7.4.1. Site I: Frontal Migraine Headaches

##### Anatomy and Trigger Points

Anatomical variations in the site where the SON exits the orbit are quite frequent, including variations between contralateral regions of the same individual. Around 83% of people have a supraorbital notch in the supraorbital rim, about 27% have a foramen just above the supraorbital rim, and roughly 10% present with both a notch and a foramen. When both are present, the SON usually divides intraorbitally and each of the two main branches leaves the orbit through separate sites, one through the notch and the other through the foramen [149,150].

The supraorbital notch, when present, tends to be situated roughly 25 mm lateral from the midline, although some variation can occur [151]. Out of the cases presenting with a notch, about 86% associate a caudal fascial band, which, according to Fallucco et al., can either be singular (type I), partially bony with spicules on the insertions of the band (type II) or a septum separating a nervous branch from the main neurovascular bundle (type III) [136]. When a foramen is present, its average distance from the midline is approximately 31 mm, though this measurement is highly variable between sexes, races and even from one side to the other in the same person [152,153,154].

After passing through either the supraorbital notch or the foramen, the supraorbital nerve (SON) usually divides into a deep and a superficial branch. The deep branch lies roughly 0.56 mm to the lateral side of a vertical line that runs through the medial limbus and it appears to be more prone to compression at the inferolateral border of the corrugator supercilii muscle (CSM), where the CSM interdigitates with the orbicularis oculi muscle [155,156]. As the branches move upward, one or both of them pass directly through the CSM in around 78% of individuals. This anatomic route likely contributes to nerve compression in this area. Further cranially, the horizontal fibers of the CSM blend with the vertical fibers of the frontalis muscle, and the opposing pull of these muscle groups can compress one or both branches of the SON [156,157,158].

The supratrochlear nerve (STN) exists in the orbit more medial than the SON through a notch or, extremely rarely, a foramen, which is usually not a trigger point. The STN tends to divide into two branches in the retro-orbicularis fat pad and one or both of these branches can also pass through the CSM, which may serve as a trigger or compression point. In most individuals (about 84%), both STN branches enter the CSM roughly 18.8 mm lateral to the midline. Further superiorly, the branches can be compressed at the level where the CSM interdigitates with the frontalis muscle [157,159,160].

##### Transpalpebral Decompression

When combined with blepharoplasty, standard markings are made, with the most caudal supratarsal crease outlined first. If blepharoplasty is not planned, the incision is limited to the medial half of the caudal crease. Following local infiltration, an incision is made through the skin and orbicularis oculi muscle and dissection proceeds superiorly toward the orbital rim in the plane between the orbicularis and orbital septum. The corrugator supercilii and depressor supercilii muscles are identified and excised using the electrocautery. A large branch of the supraorbital nerve is commonly visualized within the corrugator, which is carefully dissected and preserved during the excision of the muscle. The fascia over the supraorbital notch is released or, if a foramen is present, deroofed with an osteotome. The procerus muscle’s lateral fibers are partially resected to expose the supratrochlear nerve. A small autologous fat graft harvested from the medial fat pad of the upper eyelid is placed in the space left behind after corrugator excision and sutured to the periosteum to prevent caudal displacement. Lastly, skin closure is performed with a running subcuticular suture [161,162,163].

##### Endoscopic Decompression

Five endoscopic ports are typically used, but patients with a broad forehead may require six incisions, with the extra two paramedian ports placed approximately 0.5 cm posterior to the hairline. Additional temporal incisions are made as needed for decompression of the zygomaticotemporal branch of the trigeminal nerve, when there is a combination of temporal and frontal migraine headaches, which is most often the case. Dissection begins through the temporal incision, carried to the plane superficial to the deep temporal fascia. An endoscopic access device (EAD) is inserted, and subperiosteal dissection proceeds medially to connect all ports. In most cases, the second incision lies over the periosteum near the temporal ridge rather than the temporalis fascia. This incision is extended down to the periosteum and the second EAD is then inserted and subperiosteal dissection is performed medially toward the midline incision. A third, midline incision is subsequently created, allowing insertion of another EAD. The procedure is repeated on the contralateral side, beginning laterally and proceeding medially, after which the dissected planes are connected using a periosteal elevator. Then, the arcus marginalis and periorbita are released along the supraorbital rim, and the corrugator supercilii, depressor supercilii, and lateral fibers of the procerus muscles are resected as needed to decompress the supraorbital and supratrochlear nerves. Supraorbital foramina, when present, are unroofed with a 2 mm osteotome. Autologous fat, previously harvested from the zygomatic region during dissection, is used to fill the muscle bed and prevent postoperative adhesion. A fascial suspension suture is placed bilaterally to reposition the brow along a superolateral vector, achieving an aesthetic lifting effect [163].

A modified endoscopic approach, described by an Italian group, involves an endoscopically assisted selective myotomy rather than full muscle resection. In this technique, the corrugator and procerus muscles are sectioned through three parallel incisions placed between and lateral to the supraorbital and supratrochlear nerves, requiring only a single midline scalp incision and typically performed under local anesthesia [164,165].

##### Corrugator Denervation

Corrugator denervation represents an alternative to the endoscopic or transpalpebral resection techniques. Instead of excising the muscle, this method eliminates its motor input, leading to functional paralysis and subsequent atrophy while preserving the anatomic structure. Complete denervation requires interruption of both the lateral and medial motor supplies to the corrugator supercilii. The lateral approach involves a small incision at the inferior margin of the eyebrow. Subcutaneous and subperiosteal dissection is performed and the temporal branches of the facial nerve entering the muscle lateral to the supraorbital notch are identified and divided. The medial approach is performed through a blepharoplasty-type incision, where a periosteal window is created above the medial canthus at the frontonasal and frontomaxillary sutures. This exposes and allows transection of the medial rami of the zygomatic or buccal branches of the facial nerve, which innervate the corrugator and, if extended cephalically, the procerus muscle. Although effective in reducing muscle contractility and nerve compression, the procedure may occasionally result in hypoesthesia or anesthesia over the nasal bridge due to inadvertent injury to the infratrochlear nerve filaments [166,167].

#### 7.4.2. Site II: Temporal Migraine Headaches

##### Anatomy and Trigger Points

The zygomatic nerve is a division of the maxillary nerve (V2), which originates from the trigeminal nerve. It divides intraorbitally into the zygomaticotemporal and zygomaticofacial branches. The zygomaticotemporal nerve passes through the malar bone to enter the temporal fossa deep to the temporalis muscle, whereas the zygomaticofacial nerve emerges at the anterolateral surface of the cheek. While the zygomaticofacial branch rarely serves as a trigger site, the zygomaticotemporal branch is among the most frequent temporal migraine trigger points [168].

Anatomical studies indicate that the zygomaticotemporal nerve remains between the temporalis muscle and temporal bone in about 50% of individuals, traverses a short intramuscular segment in 22%, and follows a longer, tortuous intramuscular path in 28%, in which cases the intramuscular route is the first potential compression point [169]. It then courses superficially and pierces the deep temporal fascia, typically emerging from the fascia approximately 17 mm lateral and 6.5 mm superior to the lateral canthus, which is considered to be a second trigger point. Three accessory branching patterns have been identified: branches adjacent to, lateral to, or superior to the main trunk [170,171,172].

##### Endoscopic Decompression

The zygomaticotemporal nerve can be accessed through a variety of incisions, depending on the surgeon’s preference and whether an endoscopic or direct open technique is employed. In the standard endoscopic approach, two radial temporal incisions are made approximately 7 cm and 10 cm from the midline, positioned about 0.5 cm posterior to the temporal hairline. Dissection proceeds in the plane immediately superficial to the deep temporal fascia, extending medially toward the lateral orbital rim. Once the nerve has been identified, any accompanying vessels are isolated and cauterized. The nerve is then either decompressed by releasing the surrounding fascial bands or avulsed in a controlled manner by traction, ensuring removal of all visible branches [173].

##### Direct Lateral Canthal Approach for Decompression

A simplified, direct approach through a short lateral canthal extension incision has also been described. After local infiltration with anesthetic and epinephrine, a 0.5–1 cm incision is made and deepened to the level of the periosteum at the lateral orbital rim. Dissection continues laterally in a subperiosteal plane to expose the zygomaticotemporal nerve and its adjacent vessel just below the rim. The nerve is grasped and avulsed via traction neurectomy, followed by hemostasis. The adjacent vessel is cauterized after avulsion to minimize the risk of thermal nerve injury. To ensure complete denervation, the surgeon sweeps under the lateral orbital rim with a right-angle dissector to check for and eliminate any accessory branches [174].

##### Temporal Approach for Decompression

Another variation utilizes a deeper temporal approach. Following incision and subperiosteal dissection, the deep temporal fascia is opened down to the inferior temporal septum to enter the inferior temporal compartment. The nerve is carefully identified and freed from any potential points of entrapment within the muscle or fascial layers. When patients report pulsatile pain corresponding to vascular contact, sentinel vessels are cauterized. Care is taken to preserve the temporal branches of the facial nerve, which are incorporated into the elevated flap. These techniques collectively aim to relieve migraine symptoms by eliminating mechanical or vascular irritation of the zygomaticotemporal nerve while minimizing nerve injury and aesthetic complications [175,176,177].

##### Transpalpebral Decompression

In the transpalpebral approach for zygomaticotemporal nerve decompression, the procedure begins through the same upper eyelid incision used for corrugator muscle resection, which can be extended laterally into a horizontal periorbital crease if additional exposure is needed. Dissection proceeds laterally from the orbital rim toward the temple, elevating the superficial temporal fascia off the deep temporal fascia. The zygomaticotemporal nerve, which initially runs beneath the deep temporal fascia, is carefully identified at the point where it pierces the fascia, often in proximity to the sentinel vein and the temporal branch of the facial nerve. Once the nerve is visualized, the surrounding fascial structures are released to decompress it. If the nerve exhibits multiple branches or fascial decompression is not technically feasible, controlled avulsion of the nerve is performed instead. Hemostasis is achieved, and the incision is closed in layers. This approach allows simultaneous management of both frontal and temporal trigger points through a single transpalpebral access [175,178,179].

#### 7.4.3. Site III: Rhinogenic Migraine Headaches

##### Anatomy and Trigger Points

Rhinogenic headache refers to a headache or facial pain syndrome caused by mucosal contact points within the nasal or sinus cavities, occurring in the absence of inflammatory sinonasal disease, purulent discharge, polyps, masses, or mucosal hyperplasia [180]. Some studies have reported a significantly higher prevalence of mucosal contact points in migraine patients, particularly between the nasal septum and middle turbinate, when compared with healthy individuals [181].

A concha bullosa is defined as a pneumatized turbinate containing a visible air-filled cavity, most often involving the middle turbinate. The morphology of its contact point with the septum may be described as either focal or broad-based. The overall prevalence of concha bullosa reported in imaging studies ranges from 25% to 35%. Septal deviations are another frequent intranasal variation, observed in about 77–80% of migraine patients and 71% of non-migraine patients [182,183]. These deviations can be classified as focal, C-shaped, or S-shaped, following the system described by Guyuron [184]. Nasal spurs, which often originate from the vomer, occur in roughly 30–45% of both migraine and non-migraine patients [182,183,185].

However, larger imaging-based studies have challenged these observations, with no significant differences in the prevalence of contact points or other anatomic variations, such as septal deviation, concha bullosa, or mucosal swelling. The most frequent sites of contact were between the septum and the middle turbinate (54%), followed by the inferior turbinate (43%), while the superior turbinate contacts were rare (2%) [177]. Thus, although mucosal contact points are common (up to 87% of migraine patients), they rarely serve as the sole cause of headache. Instead, they are thought to act as secondary or amplifying factors that, under certain physiological or environmental conditions (such as mucosal swelling, infection, allergies, or barometric changes), may transform a silent contact area into an active pain trigger. The highly variable presentation reflects the dynamic nature of mucosal congestion and individual susceptibility rather than a fixed anatomical defect [182,186].

##### Septoplasty and Turbinoplasty/Turbinectomy

Septoplasty begins with an L-shaped incision, typically placed on the left side for right-handed surgeons. The mucoperichondrial flap is elevated on the left side, followed by careful incision through the septal cartilage to preserve the contralateral mucoperichondrium. Dissection continues posteriorly and caudally along the vomer and perpendicular plate of the ethmoid bone until the septal cartilage is freed. The deviated portions of cartilage and bone are excised, leaving a stable L-shaped strut measuring approximately 20 mm anteriorly and 10 mm caudally. The straight segment of cartilage is repositioned, and the mucosal incision is closed [187,188]. Endoscopic septoplasty offers enhanced visualization and access to deep nasal structures, enabling precise correction of septal deformities through minimal incisions and limited flap elevation while preserving optimal exposure of the affected area [189].

If inferior turbinate hypertrophy is present, the turbinates may be outfractured or conservatively reduced. In cases of septal bullosa or concha bullosa, the bulging or medial wall is removed to eliminate contact points while preserving functional turbinate tissue. The procedure concludes with careful hemostasis, conservative turbinectomy if required, and placement of stents to maintain septal alignment and minimize postoperative irritation. Turbinectomy must be carried out with caution, as overly aggressive resection of the turbinates can lead to postoperative nasal dryness and impaired mucosal function [190,191,192].

#### 7.4.4. Site IV: Occipital Migraine Headaches

##### Anatomy and Trigger Points

Six potential entrapment sites of the greater occipital nerve (GON) have been identified along its course. The first compression point occurs at the inferior border of the obliquus capitis inferior muscle, where fascial bands between the muscle and the nerve may produce constriction, approximately 20 mm lateral and 77 mm caudal to the external occipital protuberance (EOP) [193]. The second site is located at the deep entry of the GON into the semispinalis capitis muscle, while the third is at its superficial emergence from this muscle [194,195].

The fourth potential compression point corresponds to the entrance of the GON into the trapezius muscle, commonly called the trapezial tunnel, approximately 24 mm lateral and 21 mm caudal to the EOP. The fifth site occurs where the nerve emerges from the tendinous insertion of the trapezius along the nuchal line [188]. Finally, the sixth potential site is at the intersection of the GON with the occipital artery, which may appear either as a simple crossing or as a helical intertwining extending up to 42 mm lateral to the EOP [196].

##### Open Decompression

The procedure begins with marking the midline and occipital hairline while the patient is in a sitting position. After induction of general anesthesia and prone positioning, a 4–5 cm midline incision is made from the hairline to the occipital protuberance. Dissection proceeds through the subcutaneous tissue and trapezius muscle to expose the midline raphe and identify the vertical fibers of the semispinalis capitis muscle. The incision is carried down to the midline raphe and then extended 0.5–0.75 cm laterally on each side, preserving a 1–1.5 cm segment of the central raphe intact. The greater occipital nerve (GON) is then located just deep to the trapezius, isolated with blunt dissection, and followed along its course. If the third occipital nerve is encountered and large enough, it is decompressed or transected and buried in the semispinalis muscle [197,198,199,200,201].

The semispinalis muscle is separated from the midline raphe and a segment of its fibers medial to the GON is excised to relieve compression. After exiting the semispinalis capitis, the greater occipital nerve ascends obliquely upward and laterally beneath the trapezius toward the so-called trapezoid tunnel. The typical site of contact with the occipital artery is located just before the nerve enters this aponeurotic band. In this region, the artery may course above, below, or even intertwine with the nerve, although it is most commonly positioned beneath it. When a kinked or tortuous artery is identified, it should be removed to alleviate arterial compression of the nerve. Further dissection along the GON often reveals constricting fibrous bands that may visibly flatten the nerve. These are incised above and below the nerve until it is fully released and free from surrounding tissue. The overlying trapezial fascia is also opened laterally, and any fascial or vascular structures compressing the nerve are released. Branches of the occipital artery crossing or entwined with the nerve are ligated and divided. When present, tattoo markings from Doppler localization guide the identification of vascular compression points [199,200,201].

A subcutaneous flap, based caudally and positioned superior to the nerve, is then elevated and passed beneath the GON to shield it from surrounding muscle tissue. The flap is anchored to the midline raphe to maintain decompression. A drain is placed through a separate incision, and layered closure of the subcutaneous tissue and skin is performed. The incision is dressed with topical antibiotic ointment before extubation and recovery [197,198,199,200,201].

##### Endoscopic Decompression

A 3–4 cm midline incision is made within the occipital hairline, just below the external occipital protuberance. Dissection proceeds through the trapezius, where the oblique fibers are identified, and any encountered third occipital nerve may be avulsed if necessary. Deeper exposure reveals the vertical fibers of the semispinalis capitis muscle. Blunt dissection is carried laterally between the semispinalis and the trapezius fascia until the GON is visualized approximately 1.5 cm lateral and 3 cm inferior to the occipital protuberance. To decompress the GON, a 1 cm^2^ portion of the semispinalis muscle medial to the nerve is excised, releasing it from intramuscular compression. Proximal decompression is completed by freeing the nerve from the overlying fascial bands, while distal decompression involves releasing the trapezius tunnel under direct vision. The GON is then separated from the occipital artery through a small oblique incision medial to the ear and above the mastoid region. The artery is carefully dissected away from the nerve, cauterized, and excised along its exposed length to prevent pulsatile compression. Any additional nerve branches that cannot be adequately decompressed are cauterized. To protect the released nerve and minimize postoperative scarring, laterally and caudally based subcutaneous fat flaps are mobilized, passed beneath the GON, and sutured to the midline raphe. In some cases, nerve wraps may be applied for additional cushioning. A suction drain is placed before closure. If present, the lesser occipital nerve (LON) is also addressed through decompression or traction neurectomy at its emergence along the posterior border of the sternocleidomastoid muscle [202,203].

#### 7.4.5. Site V: Auriculotemporal Migraine Headaches

##### Anatomy and Trigger Sites

The auriculotemporal nerve arises from the mandibular division of the trigeminal nerve (V3) after its exit through the foramen ovale. The first point of compression is sometimes identified right in relation to the mandibular condyle and the temporomandibular joint capsule [204]. In the temporal region, it courses superficially, lying within the temporoparietal fascia inferiorly and within the subcutaneous tissue superiorly. The terminal portion of the auriculotemporal nerve, known as the superficial temporal branch, exhibits marked anatomical variability. Although traditionally described as a single trunk dividing into smaller branches, studies have shown that this configuration occurs in only about half of cases, with others displaying multiple trunks, diffuse branching, or complex plexiform and looping patterns in the preauricular and temporal regions [205,206].

Three other compression points have been identified along the course of the auriculotemporal nerve: two caused by consistent preauricular fascial bands crossing the nerve, and a third at its intersection with the superficial temporal artery. Clinically, however, most migraine patients localize pain to the neurovascular intersection rather than to the fascial band sites [207].

##### Decompression Surgery

A small incision is made within the hair-bearing scalp at the base of the sideburn, and careful dissection is carried down to expose the auriculotemporal nerve and the accompanying superficial temporal artery. Once identified, the artery is ligated and divided, and the nerve is mobilized over a sufficient length to allow its repositioning. A small opening is then created in the deep temporal fascia, and a pocket is developed within the temporalis muscle. The transected end of the nerve is gently placed and secured within this intramuscular pocket to prevent neuroma formation and reduce postoperative irritation. If the nerve cannot be adequately separated from the accompanying artery, it has been recommended that the entire neurovascular bundle be ligated and excised. In smaller accessory sites, a shorter incision is sufficient and the targeted vessel is ligated or cauterized, and if a small nerve branch is encountered, it is simply avulsed without inserting it into the muscle [208,209].

#### 7.4.6. VI: Lesser Occipital Migraine Headaches

##### Anatomy and Trigger Points

The lesser occipital nerve (LON) emerges at or through the posterior border of the sternocleidomastoid muscle, representing the first potential compression site. It may intersect the occipital artery in about half of individuals, forming a second possible entrapment point, usually as a simple crossing but occasionally as a helical intertwining. A third site, present in roughly 20% of cases, involves a fascial band along its course, lateral to the midline and inferior to the external auditory canal [210,211].

The third occipital nerve (TON) consistently emerges through the semispinalis capitis muscle, forming its primary potential compression site about 13 mm lateral to the midline and 60 mm caudal to the external auditory canal, which is approximately 3 cm below the GON [197]. Other potential trigger points are the piercing point of the splenius capitis and the trapezius muscles [212].

##### Decompression

A small horizontal incision, approximately 1.5–2 cm long, is planned at the confirmed point. The incision is made through the subcutaneous tissue, and careful blunt dissection is carried out to expose the underlying trapezius fascia, where the nerve and associated vessel are typically located. Once identified, the vessel is dissected, cauterized, and divided. If a fascial compression point is found, it is released; otherwise, the nerve is isolated, dissected, and transected to approximately 1.5 cm in length. A small intramuscular pocket is then created within the trapezius to bury the proximal nerve stump, minimizing the risk of neuroma formation. The nerve is secured within the muscle pocket by passing a suture through the muscle, looping it around the nerve, and tying it so the nerve end retracts into the muscle [201,213].

Alternatively, if the surgery is performed for both GON and LON decompression, the primary midline incision in the GON decompression surgery may be extended laterally, or a separate incision is made over the posterior border of the sternocleidomastoid muscle to access the LON. Dissection proceeds through the occipital, trapezius, and portions of the sternocleidomastoid muscles to expose and free the nerve. When both GON and LON trigger points are present, a medial incision can be employed to allow simultaneous isolation and decompression of both nerves and the accompanying occipital vessels [214].

Although isolated LON involvement is less common than GON-related compression, its role may be underrecognized. Patients with incomplete relief after GON-targeted decompression or botulinum toxin therapy should be evaluated for concurrent LON entrapment [211].

### 7.5. Autologous Fat Grafting

Fat grafting (lipofilling) has shown promising benefits in the management of neuropathic pain, particularly in cases resistant to conventional therapies. As a minimally invasive and safe procedure, it offers the potential for significant symptom relief with minimal side effects. The therapeutic effect of fat grafting is believed to be linked to the biological activity of adipose-derived stem cells (ADSCs), which are present in the grafted fat. These cells can secrete anti-inflammatory cytokines, promote nerve regeneration, and modulate the local inflammatory environment. Experimental studies suggest that ADSCs may also differentiate into Schwann-like cells and support nerve repair by enhancing neurite outgrowth and reversing nociceptive hypersensitivity. Additionally, the mechanical properties of the grafted fat may contribute to pain relief by increasing scar softness and releasing entrapped nerves, thereby reducing abnormal nerve stimulation [215,216,217].

The technique, as described by Guyuron et al., consists of harvesting 10–30 cc of fat from the abdomen or lateral thigh using a 2 mm cannula and a 10-cc syringe, preparing the fat using the Coleman technique (centrifuged at 3000 rpm for 5 min), and injecting it with 1-cc syringes using 0.7–0.9 mm blunt cannulas. Injection sites were selected based on anatomic knowledge of the relevant trigeminal and cervical nerve branches, taking into account the region of pain indicated by the patient. The injections were delivered subcutaneously, subfascially, and deep to the nerve, following its course, in a fanning motion around the identified nerve trigger sites. The volume of fat injected per affected site was standardized as follows: 1 cc for site I, 2 cc for sites II, V, and VI, and 2.5 cc for site IV [218,219].

Autologous fat grafting can be performed during primary migraine surgery either as an alternative to or in combination with fat pad placement at site I and site IV to reconstitute tissue volume following myectomies and create a protective buffer around the nerves, thereby reducing migraine triggers. Patients who do not respond to primary decompression surgery are considered for fat injection at least 3 months postoperatively, once inflammation has subsided and symptoms have stabilized. If localized residual pain persists after primary surgical decompression, evaluation and possible decompression of a secondary migraine trigger site may be warranted. For diffuse residual pain or incomplete relief following surgery, fat injection is the preferred treatment. Repeat fat injection may be performed in cases of partial improvement; however, if two rounds fail to achieve satisfactory relief, neurectomy is indicated [218].

## 8. Recent Advances in Migraine Research

In addition to the established therapies used in the management of migraine, recent years have witnessed a significant intensification of research efforts aiming to develop new compounds and technologies capable of improving clinical outcomes.

Recent work in migraine research has broadened therapeutic exploration beyond the CGRP (calcitonin gene–related peptide) pathway. Investigators are now examining a wider set of molecular and neurophysiological targets involved in trigemino-vascular activation, sensory processing and central modulation of pain. CGRP monoclonal antibodies and gepants remain the most advanced mechanism-based treatments. However, growing evidence shows that other peptide systems, such PACAP (Pituitary Adenylate Cyclase–Activating Polypeptide) and amylin, also play roles in migraine biology and may influence differences in treatment response. Research on ion-channel modulation, including TRP channels (Transient Receptor Potential Channels), K-ATP (ATP-Sensitive Potassium Channels), BK-Ca (Big Potassium Calcium-Activated Channels), and ASICs (Acid-Sensing Ion Channels), highlights the importance of neuronal excitability and peripheral sensitization in triggering and sustaining attacks. Interest is also increasing in endocrine, hypothalamic, vascular, and neuroimmune pathways. These directions support the view that migraine is a multidimensional disorder, especially in patients with hormonal triggers, circadian patterns, or progression to chronic migraine [123,220,221,222,223,224,225,226,227,228]. Table 4 provides an overview of recent therapeutic developments in migraine, including both established and emerging mechanisms.

Novel surgical visualization technologies, such as high-definition exoscopes and robot-assisted digital microscopy, have been increasingly adopted in cranial nerve procedures, particularly microvascular decompression for trigeminal neuralgia. In a retrospective study on 135 patients, Kramer et al. compared endoscope, exoscope, and microscope visualization during microvascular decompression for trigeminal neuralgia. Clinical outcomes were equivalent across microscope-, endoscope-, and exoscope-assisted procedures; however, endoscopic cases had shorter operative times, and both the endoscope and exoscope exhibited fewer postoperative complications than the microscope. Notably, the endoscope and exoscope identified two or more compressive vessels in 9–13% more cases and reported an absence of vascular compression in 7–9% fewer cases compared with the microscope, while also providing enhanced ergonomics and superior visualization for surgical trainees [260].

Rossini’s experience indicates that robot-assisted exoscopic visualization is a feasible and safe alternative for microvascular decompression in trigeminal neuralgia, providing stable stereoscopic imaging, improved surgeon ergonomics, reliable identification of neurovascular compression, and a meaningful reduction in operative time compared with traditional microscopic techniques [261].

Although these platforms have not yet been systematically evaluated in migraine surgery, their demonstrated advantages in peripheral nerve and skull-base operations include enhanced visualization, improved ergonomics and more precise instrument handling [262].

Another novel therapeutic direction is represented by the utilization of alternative forms of botulinum toxin other than OnabotulinumtoxinA. A study reported by Ion et al. investigates the efficacy of incobotulinumtoxin A as a prophylactic treatment for chronic refractory migraine. Conducted on 61 patients, the results revealed that of Incobotulinumtoxin A significantly reduces the frequency of migraine episodes and headache days, with a >50% reduction observed in three quarters of participants. The mean number of drug doses also decreased substantially. Patients reported a sustained response over time, with improvements noted for those with chronic migraine, tension-type headache, and medication overuse headache. Treatment was generally well-tolerated, with minimal adverse events [263]. A recent study on 112 chronic migraine patients compared AbobotulinumtoxinA (AboBoNT-A) with OnabotulinumtoxinA (OnaBoNT-A) over one year. Both treatments reduced migraine-related disability, with similar improvements between groups. The findings suggest that AboBoNT-A may be an effective option, but larger studies are needed to confirm its long-term value [264]. Also, DaxibotulinumtoxinA is mentioned for potential efficacy in patients with concurrent oromandibular dystonia and chronic migraine [265].

## 9. Discussion

### 9.1. Overview of Existing Literature

Headache disorders constitute a substantial source of global disability and are a frequent cause of addressing healthcare across general practice, neurology outpatient clinics and emergency departments. Headache is a common reason for outpatient care in the United States, ranking as the seventh most frequent presenting complaint in ambulatory settings. Historically, the study and tracking of headache disorders has been limited by inconsistent terminology and diagnostic criteria, a challenge substantially reduced following the development of the International Headache Society’s first operational classification for primary headaches in 1988. Among primary headache disorders, tension-type headache and migraine are more prevalent in women, whereas cluster headache shows a higher incidence in men, reflecting well-documented sex differences in headache epidemiology [266,267,268,269].

Migraine is a common neurological disorder, affecting approximately 12% of the general population. Annual prevalence rates are higher in women, reaching up to 17%, compared with about 6% in men. Prevalence rises during puberty, reaches a peak between 35 and 39 years of age, and subsequently declines with advancing age, particularly after menopause [22].

Surgical treatment of migraine was explored as early as the beginning of the 20th century by Dr. Harvey Cushing, widely regarded as the father of modern neurosurgery. However, many of these early surgical attempts yielded inconsistent or unsuccessful outcomes and were never formally published [270,271].

The initial therapeutic interaction between plastic surgery and headache management can be explained by the substantial demographic overlap between patients with migraine and other headache disorders and the population commonly seeking aesthetic surgical procedures, in whom headache complaints were frequently encountered.

Early research into migraine surgery introduced by Dr. Bahman Guyuron was inspired by observations from two patients who reported complete resolution of their migraine headaches following forehead rejuvenation procedures. Subsequently, a retrospective analysis of 314 patients who underwent forehead rejuvenation demonstrated improvement or complete elimination of migraine symptoms in 31 of the 39 patients with a preoperative history of migraine headaches. Through further studies, surgical treatment was introduced as a viable treatment in migraine headaches, with efficacy, longevity and safety of results, also with significant socioeconomic benefit, reducing both direct and indirect healthcare costs and increasing patient participation in daily activities [25,272,273]

It is essential to clearly define and understand the contemporary role of surgical interventions within the comprehensive, multimodal management of patients with migraine. Surgical treatment does not constitute a first-line therapeutic option; rather, it represents an adjunctive strategy reserved for carefully selected patients in whom optimized pharmacologic therapy and evidence-based non-pharmacologic interventions have failed to achieve adequate disease control.

A structured escalation in migraine care ensures patients are optimally treated medically before referral for surgical intervention. Current evidence and guideline documents support, after an adequate diagnosis implementation of a stepwise, multimodal therapeutic strategy.

Migraine diagnosis must be confirmed according to ICHD-3 criteria, with exclusion of secondary headache disorders. Comorbidities that negatively affect treatment response, particularly medication-overuse headache (MOH), sleep disorders, psychiatric disease and hormonal or metabolic factors must be identified and actively managed. A structured headache diary is mandatory to document frequency, severity, disability, medication use and attack onset patterns [3,274].

Evidence-based abortive therapy must be appropriately selected, dosed and timed, with early administration during attacks. First-line agents include NSAIDs and acetaminophen, followed by migraine-specific therapies (triptans, ditans, gepants) for moderate–severe attacks. In patients with nausea, vomiting or gastric stasis, non-oral formulations (nasal, rectal, injectable) are required to ensure bioavailability. Abortive medication frequency must be limited to prevent MOH, as uncontrolled overuse invalidates assessment of treatment refractoriness [116,275,276,277,278].

Preventive treatment is indicated for patients with frequent attacks or significant disability and must include adequate trials of guideline-supported agents (beta-blockers, antiepileptics, antidepressants, calcium channel blockers and sartans). Each preventive should be titrated to an effective or maximally tolerated dose and continued for a minimum of 8–12 weeks, with clinical success defined as a ≥50% reduction in attack frequency, severity, or duration [279,280].

In patients with persistent disability despite conventional preventives, escalation to CGRP-pathway therapies (monoclonal antibodies or preventive gepants) is supported by high-level evidence and contemporary guidelines. These agents target trigeminovascular signaling without vasoconstriction and represent a standard component of modern preventive care prior to considering a patient as medically refractory [281,282].

For patients meeting criteria for chronic migraine, onabotulinumtoxinA administered according to the PREEMPT protocol (155–195 U every 12 weeks) constitutes an established preventive therapy. Adequate assessment requires at least two treatment cycles, with documentation of response or failure and correction of MOH prior to initiation [283,284].

Multiple reports also exist on sphenopalatine ganglion block for managing facial pain conditions, including trigeminal neuralgia, acute migraine, post-dural puncture headache, postoperative pain after adenotonsillectomy and perioperative analgesia for cleft palate repair. Delivering anesthetics into this space can reduce complications and limit the need for systemic steroids, while combining vasoconstrictors with local anesthetics during paranasal sinus surgery has been shown to substantially decrease intraoperative bleeding. As a result, multiple techniques for administering agents into the pterygopalatine fossa have been developed and investigated in recent years. The clinical literature remains heterogeneous regarding the efficacy of with a mixture of small randomized controlled trials and observational studies. Results regarding efficacy in acute migraine, chronic migraine, and post–dural puncture headache have been mixed, and methodological variability (technique, anesthetic agent, dosing and comparator design) limits generalizability. Reported reviews continue to categorize SPG block as a promising but incompletely validated intervention. Taken together, emerging imaging and histological data substantiate the mechanistic rationale for both non-invasive and percutaneous SPG-targeted treatments, while simultaneously underscoring that the choice of approach affects treatment specificity and potential clinical outcomes [139,148,274,285,286,287,288].

Trigger-site migraine headache surgery should be considered only in a carefully selected population of patients with persistent, disabling migraine who have failed comprehensive, guideline-concordant non-surgical management. Candidates must have a confirmed diagnosis of migraine according to ICHD-3 criteria, with secondary headache disorders excluded and major confounding factors (particularly medication-overuse headache) identified and appropriately corrected prior to designation of medical refractoriness [3,289].

The role of the surgeon within the multidisciplinary care team extends beyond technical execution and includes rigorous evaluation of surgical candidacy, precise correlation of clinical symptomatology with regional anatomy and a comprehensive understanding of both the potential benefits and inherent limitations of operative treatment. Accordingly, surgical options occupy a clearly defined position as advanced-line interventions, integrated into a structured therapeutic algorithm designed to optimize patient outcomes while minimizing procedural risk.

Surgical candidacy requires documented failure, intolerance or contraindication to optimized acute and preventive pharmacologic therapy. This includes appropriate trials of evidence-based oral preventive agents, escalation to CGRP-pathway therapies and, in patients meeting criteria for chronic migraine, an adequate trial of onabotulinumtoxinA administered according to the PREEMPT protocol. Acute therapy must have been optimized with correct timing, dosing and route of administration, including non-oral formulations when gastrointestinal symptoms limit bioavailability [24,134,275,281,290].

In addition to medical refractoriness, patients should demonstrate clinically meaningful disease burden, such as frequent severe attacks associated with significant impairment of occupational, educational or social functioning [291].

Surgical consideration depends on the identification of reproducible, anatomically consistent peripheral trigger site(s), supported by headache diaries, symptom localization and focused physical examination findings. These features distinguish patients with a plausible peripheral contribution to migraine initiation from those with predominantly diffuse central chronicization who are less likely to benefit from surgical intervention. Gfrerer et al. introduced the “PAINS” decision tool as a pragmatic, clinic-facing framework to standardize candidate screening and trigger-site localization in patients being evaluated for migraine trigger-site surgery. PAINS operationalizes five core elements: (P) a discrete, reproducible pain point identifiable with a single finger at attack onset; (A) an appropriate symptom constellation consistent with the suspected trigger phenotype; (I) improvement with injectables (local anesthetic nerve blocks during active pain or botulinum toxin A when pain is absent), used as supportive evidence for peripheral trigger involvement; (N) a neurologist-confirmed diagnosis to reduce misclassification and ensure appropriate exclusion of alternative headache disorders and (S) sketch matching, in which patient-drawn pain onset and radiation patterns are compared with characteristic distributions to aid trigger-site mapping [26].

The patients who are most likely to benefit from surgery are patients whose headaches persist despite optimized pharmacologic therapy and who meet standard diagnostic criteria, who have a clear identifiable trigger site with positive response to diagnostic injections [292,293,294].

In a prospective randomized study conducted by Guyuron et al., surgical deactivation of migraine trigger sites resulted in clinically meaningful improvement in the majority of treated patients. Among patients who underwent surgery after positive BT-A screening, 92% achieved at least a 50% reduction in migraine frequency, duration, or intensity. Complete elimination of migraines occurred in 35%, while 57% experienced significant improvement over a mean follow-up of approximately 13 months. In contrast, improvement was uncommon in the control group (15.8%), with no cases of migraine elimination. Significant improvements were also observed in validated patient-reported outcomes, along with substantial reductions in healthcare utilization and work absenteeism. Notably, clinical benefit increased progressively over time, arguing against a short-lived placebo response. Surgical complications were largely sensory or inflammatory in nature, including temporary anesthesia at operative sites (universal), nasal dryness, rhinorrhea, scalp pruritus, and minor alopecia. Infectious complications were uncommon (sinus infection ~3%) and were mitigated with modification of postoperative antibiotic protocols. Importantly, no permanent neurological deficits or life-threatening complications were reported, and most adverse effects resolved spontaneously.

Judging by the results of this study, patients should be counseled that most will experience meaningful migraine reduction rather than complete elimination and that improvement may continue gradually over several months following surgery. Transient sensory changes (numbness, itching), esthetic alterations, and nasal symptoms are common and should be anticipated. Clinicians should emphasize that temporary side effects are expected, while serious or permanent complications are rare. Clear preoperative counseling regarding the possibility of incomplete response, particularly if not all trigger sites are identified, is essential to set realistic expectations.

Despite growing interest in surgical decompression as a treatment for refractory migraine, the procedure remains controversial, and many neurologists are understandably hesitant to recommend it. This caution reflects persistent uncertainty in the existing evidence base. Much of the supporting literature consists of small, single-center studies, retrospective analyses, or trials conducted by highly specialized surgical teams, which may limit generalizability. Additionally, heterogeneity in patient selection criteria, surgical techniques, and outcome measures complicates interpretation and comparison across studies. Randomized controlled trials evaluating migraine decompression surgery remain limited in number, and while some demonstrate statistically significant improvements in headache frequency or severity, concerns persist regarding potential placebo effects, particularly given the invasive nature of the intervention. Blinding is inherently challenging in surgical trials, and sham-controlled designs, though valuable, have been few and subject to methodological debate. Furthermore, long-term follow-up data extending beyond several years are sparse, leaving uncertainty regarding the durability of benefit and the potential for late complications or symptom recurrence [295,296].

Reported outcomes after migraine trigger site surgery do seem to vary by anatomical location, though comparisons are complicated by differences in study design and reporting. Among the various sites, occipital decompression has shown some of the most consistent results, with several case series describing improvement or elimination rates in the 80–95% range [297].

Frontal and temporal trigger sites have also demonstrated clear benefit, but with a wider spread of reported success rates, generally ranging from about 57% to over 90%, likely reflecting differences in technique, follow-up and patient selection [298]. In contrast, evidence supporting surgery for nasal or rhinogenic trigger sites, such as septoplasty or sinus procedures, is more limited and comes primarily from smaller series. When data across trigger sites are pooled, overall benefit is observed, but the marked heterogeneity suggests that surgical outcomes depend heavily on both the specific trigger site and careful selection of appropriate patients [299,300].

To better define the role of surgical decompression in migraine management, further high-quality evidence is needed. Future studies should prioritize standardized diagnostic criteria for identifying appropriate surgical candidates, including clearer validation of peripheral nerve compression as a primary migraine driver rather than a secondary phenomenon. Additionally, larger multicenter randomized controlled trials with rigorous sham controls, standardized outcome reporting, and extended follow-up are essential to assess both efficacy and long-term safety. Improved mechanistic studies, incorporating imaging, neurophysiologic data, and biomarkers, may further clarify which patient populations are most likely to benefit. Until such evidence is available, surgical decompression should be considered cautiously and primarily within multidisciplinary care frameworks or research settings [33,301].

Comparative effectiveness studies evaluating surgical intervention against optimized, contemporary medical therapy, including CGRP-targeted treatments, are also necessary to contextualize surgical outcomes within current migraine management paradigms [302].

Patient safety in migraine headache surgery depends on a careful, step-by-step approach, as risks may arise at several points in care, from preoperative assessment through intraoperative decision-making. One of the most important safety issues is accurate identification of the migraine trigger site. Because headache localization and patient descriptions can vary, trigger sites should not be assigned based on a single clinic visit. Rather, confirmation at a second preoperative evaluation helps ensure that the planned surgical site consistently matches the onset of symptoms, reducing the risk of operating on an incorrect site and improving overall safety [280,300].

Thorough documentation within the informed consent is another important safety step. Clearly recording which nerve is planned for decompression or ablation, the side of the procedure and whether nearby vascular structures are expected to be involved helps maintain clarity for the parties involved. When Doppler examination suggests arterial involvement, it is especially important to document any plan for arterial ligation. In the same way, noting adjunctive measures, such as fat grafting or injection or corticosteroid injection, helps ensure the operative plan is clearly understood and followed intraoperatively. From a technical perspective, avoiding intra-arterial injection of local anesthetic is a key safety concern, particularly at trigger sites that overlie arterial structures. In these situations, an anesthetic substance should be infiltrated from a more distant injection site, with slow advancement toward the surgical field, which reduces the risk of intravascular injection and related complications [157,303].

Anesthetic management is critical for patient safety in migraine surgery. When possible, procedures should be performed under local anesthesia with minimal sedation to reduce exposure to general anesthesia, which is typically reserved for rhinogenic trigger sites requiring airway protection. This consideration is especially important in patients undergoing multilevel or staged interventions. Minimizing total anesthesia time by limiting repositioning, treating multiple trigger sites in one session, and using shared or minimally invasive incisions, helps reduce operative risk [273,304].

All in all, patient safety in migraine headache surgery is optimized through thorough trigger-site confirmation, proper documentation and careful anesthetic technique, aside from efficient operative planning. Together, these strategies form a cohesive safety framework that supports accurate, reproducible and patient-centered surgical care [305].

**Figure 5:**
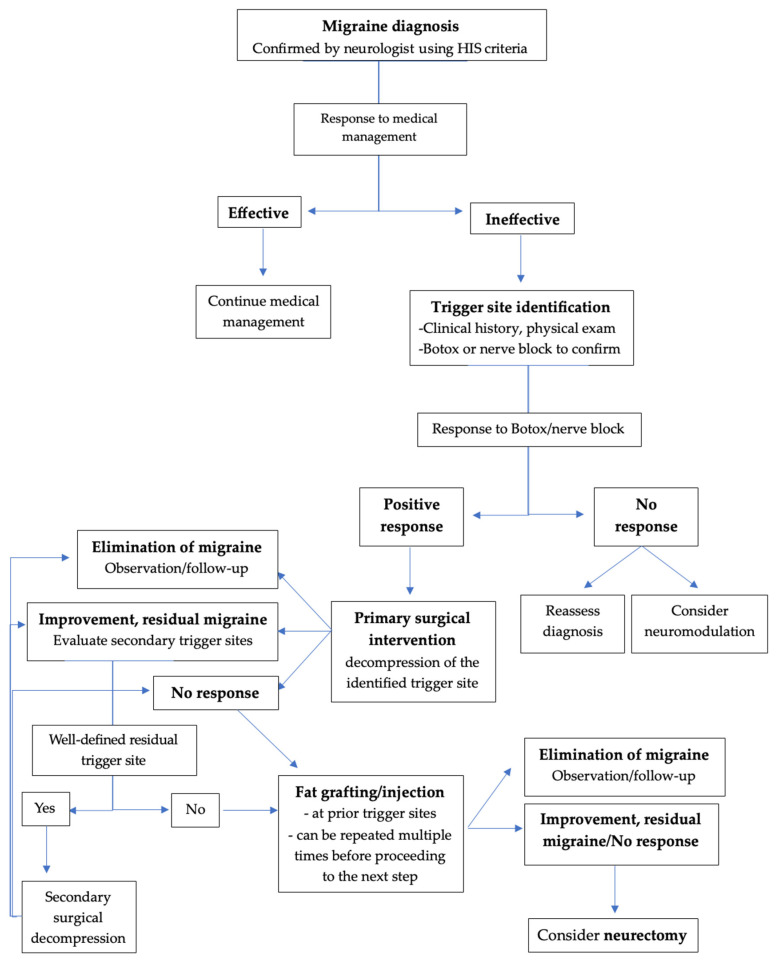
Comprehensive algorithm for migraine surgery.

A comprehensive algorithm for migraine surgery is depicted in Figure 5.

### 9.2. Critical Appraisal of the Evidence

The existing literature on migraine management reflects significant heterogeneity in study design, patient populations and outcome measures, particularly when comparing pharmacologic, interventional and surgical approaches.

High-level evidence supports the efficacy of contemporary pharmacologic therapies, including CGRP-pathway agents and onabotulinumtoxinA, which demonstrate reproducible benefit across large randomized controlled trials.

In contrast, evidence supporting interventional and surgical treatments is more variable, with outcomes strongly influenced by patient selection, diagnostic rigor and procedural technique.

For interventions such as sphenopalatine ganglion blockade, published results range from a mixture of small randomized controlled trials and observational studies, reflecting methodological variability in delivery technique, anesthetic agent, dosing, and study endpoints. While therapeutic rationale is supported by anatomical, imaging and neurophysiological data, inconsistency in trial design limits generalizability and precludes definitive conclusions.

Consensually, consideration for surgical intervention is appropriate only after there is documented evidence of failure, intolerance, or contraindication to fully optimized acute and preventive pharmacologic treatment, tailored to the patient’s needs and expectations. This process must include adequate trials of evidence-based oral preventive therapies, stepwise escalation to CGRP-pathway–targeted treatments, and for patients who meet diagnostic criteria for chronic migraine, a sufficient course of onabotulinumtoxinA administered in strict accordance with the PREEMPT protocol. Additionally, acute treatment must have been appropriately optimized with correct timing, dosing and adequate route of administration to ensure optimal bioavailability [24,134,275,281,290,306].

Surgical consideration depends on the identification of reproducible, anatomically consistent peripheral trigger site(s), supported by headache diaries, symptom localization and focused physical examination findings. Accordingly, the strengths of the surgical literature lie in anatomically based pathophysiological rationale, the systematic use of preoperative trigger-site localization strategies, including symptom mapping and response to diagnostic injections and a consistent emphasis on careful patient selection. At the same time, this dependence on precise trigger-site identification underscores a key limitation of surgical migraine treatment, as variability in patient-reported symptoms, overlap between trigger sites, and incomplete reproducibility of peripheral pain generators may constrain both candidacy assessment and generalizability of reported outcomes. Appropriate case selection is therefore essential for a successful surgical outcome [24,290,292,307,308].

Across recent systematic reviews and meta-analyses, extracranial “trigger-site” migraine surgery is repeatedly linked to postoperative improvements in headache outcomes among appropriately selected patients, but the certainty of evidence remains limited by study design and heterogeneity.

The meta-analysis conducted by Nagori et al. found statistically significant reductions in headache frequency and intensity after surgery, but with very high heterogeneity [290].

The PRISMA systematic review/meta-analysis by Evans et al. (prospective-only quantitative synthesis) similarly reports clinically meaningful improvements in frequency, severity, and migraine index score, but explicitly notes that meta-analyses were based on a small number of analyzable studies and included trials with high risk of bias, limiting precision and generalizability [307].

A systematic review by Henriques et al. reports broad ranges of “significant improvement” and “complete elimination” across studies, supporting that benefit is frequently observed, but the wide ranges themselves underscore heterogeneity and likely differences in selection and definitions across cohorts [292].

In synthesis, limitations repeatedly cited across the field include small sample sizes in many series, single-center experience, heterogeneity in operative techniques and trigger-site definitions, variability in outcome measures and relatively limited numbers of controlled randomized trials. In addition, the invasive nature of surgery poses inherent challenges to blinding and may contribute to concerns regarding placebo effects, while long-term durability data beyond several years remain comparatively limited. Reported outcomes also appear to vary by anatomical site, but interpretation is complicated by heterogeneity in study methodology and reporting [24,290,292,293,306,307,308,309]. Surgical decompression of peripheral migraine trigger sites clearly demonstrated high response rates in selected cohorts, particularly for occipital, frontal, and temporal trigger sites [297,299,300,307].

Additionally, emerging therapies, including new pharmacological agents, neuromodulation or surgical options like autologous fat grafting, require further analysis of their extensive clinical efficacy in order to be included in standardized therapeutic protocols for migraine headaches.

Collectively, the available evidence supports a nuanced interpretation in which surgical and interventional approaches are not first-line therapies, but rather advanced, mechanism-specific options whose effectiveness depends critically on rigorous diagnosis, precise identification of peripheral trigger sites, careful patient selection and integration within a structured multidisciplinary treatment algorithm. Peripheral nerve decompression surgery has been shown to significantly reduce migraine headache frequency and intensity in selected patient populations; however, therapeutic response is not universal and a subset of patients derives little or no benefit. This variability highlights the need for further clinical and anatomical studies to better define the mechanisms of peripheral nerve compression in migraine pathophysiology and to clarify the factors contributing to non-response to surgical treatment, with the goal of refining patient selection and improving long-term outcomes.

### 9.3. Reflexivity and Contextual Considerations

This review was conducted as a narrative synthesis, and its interpretive nature introduces inherent limitations. The information was selected and interpreted in consideration of the authors’ collective professional background, but in a comprehensive manner, aiming to cover a broad and representative body of literature relevant to this field.

Additionally, the heterogeneity of the underlying literature, including variability in diagnostic criteria, outcome reporting, and follow-up duration, limits direct comparison across studies and may influence the level of evidence. Publication bias favouring positive surgical outcomes and underreporting of negative or neutral results must also be acknowledged.

Although migraine is a common clinical condition, its surgical management remains an emerging and relatively specialized field, requiring expertise in peripheral nerve microsurgery that is not universally available across practitioners or clinical centers. The sustained involvement of the plastic surgeon as a member of the multidisciplinary migraine treatment team should be encouraged to ensure the availability of a broader range of therapeutic options.

Limited accessibility to consolidated, high-quality information further contributes to this gap and further scientific work on this topic may facilitate a more informed dissemination and understanding of surgical options within migraine care.

To overcome these limitations, this review integrates data from neurology, pain medicine, and surgical literature, emphasizes guideline-concordant medical management before surgical consideration, and explicitly addresses areas of controversy and evidentiary weakness. Nevertheless, conclusions regarding the role of surgical decompression should be interpreted within the context of evolving evidence and individualized patient assessment.

## 10. Conclusions

Migraine remains a complex and multifactorial disorder with significant functional and psychosocial impact, yet advances in both nonsurgical and surgical interventions have expanded the therapeutic strategies. A clear understanding of migraine clinical presentation enables more precise identification of trigger sites and guides the selection of targeted therapies. From a plastic surgeon’s perspective, the integration of anatomical expertise with a multimodal treatment algorithm allows for individualized care that spans conservative, diagnostic, and definitive options. Ultimately, a tailored approach that incorporates onabotulinumtoxinA injections, surgical decompression, and adjunctive methods such as lipofilling provides a comprehensive pathway towards migraine relief, improving quality of life for patients whose symptoms have often been refractory to traditional medical management.

## Figures and Tables

**Table 1:** Objectives of the Review.

Objective	Description
Integrate current concepts of migraine pathophysiology	To synthesize contemporary evidence on central neurovascular mechanisms and extracranial peripheral nerve involvement underlying migraine initiation and perpetuation.
Characterize migraine trigger sites and their anatomical basis	To describe clinically relevant peripheral trigger sites and their relationship to extracranial nerve compression, facilitating anatomically informed diagnosis and treatment planning.
Define a mechanism-based diagnostic framework	To outline structured diagnostic approaches, including headache phenotyping, trigger-site mapping and the use of adjunctive tools such as nerve blocks and onabotulinumtoxinA injections.
Review therapeutic strategies across the migraine spectrum	To evaluate pharmacologic treatments, injectable therapies, neuromodulation techniques and surgical decompression as part of a comprehensive migraine management strategy.
Clarify the role of surgery within multidisciplinary care	To clarify indications, patient selection criteria and the position of surgical decompression as an advanced, adjunctive option within a multidisciplinary migraine treatment algorithm.
Provide clinical guidance for treatment escalation and referral	To offer a practical diagnostic and therapeutic reference for plastic surgeons, neurologists, pain specialists and general practitioners involved in migraine care.

**Table 2:** Headache onset area and trigger point identification in migraines [25,26].

Site	Primary Nerves & Compression Structures	Pain Distribution & Distinguishing Features	Common Triggers
I—Frontal	Supraorbital (SON) & supratrochlear (STN) nerves; compression beneath corrugator, depressor supercilii, procerus; SON at notch/foramen; periosteal fascial bands.	Forehead/glabellar pain ± medial orbital radiation; brow heaviness or eyelid ptosis; Tinel over supraorbital rim; often later-day worsening.	Frowning, stress, eye strain; sustained frontalis/corrugator activation.
II—Temporal	Zygomaticotemporal nerve (ZTN, V2) within deep temporal fascia; compression near sentinel vein; possible fascial slit entrapment.	Posterior temple pain superior to zygomatic arch; pressure sensitivity over sentinel vein; can co-occur with preauricular pain.	Jaw clenching, bruxism, TMJ loading; external pressure at posterior temple.
III—Rhinogenic (Nasal)	Mucosal contact points: deviated septum or septal spur contacting middle/inferior turbinate or ethmoidal structures; trigeminal afferent sensitization.	Retro-orbital and midface pain; nasal obstruction; pressure behind eyes; often cyclic with allergy/hormonal variation.	Allergens, weather/barometric changes, hormonal shifts; supine position.
IV—Occipital (Greater ± Lesser/Third)	Greater occipital nerve (GON) at semispinalis capitis, trapezius aponeurosis, and occipital artery crossing; ± lesser (LON) and third occipital nerves.	Suboccipital pain radiating to vertex/forehead; scalp allodynia; Tinel 2–3 cm lateral to midline at superior nuchal line.	Neck muscle tension, heavy exercise, prolonged flexion/extension; stress.
V—Auriculotemporal (Preauricular)	Auriculotemporal nerve (ATN, V3) adjacent to superficial temporal artery (STA) and superficial temporal fascia; arterial loop or fascial bands can compress.	Preauricular/temporal pain with pulsatile or bounding quality; can mimic primary vascular headache; point tenderness anterior to tragus.	Weather/barometric shifts; mastication; external pressure (headwear).
VI—Lesser Occipital (Lateral Neck)	Lesser occipital nerve (C2–C3) at posterior border of sternocleidomastoid (SCM) and along the cervical plexus; fascial entrapment near Erb point.	Retroauricular and lateral neck pain radiating superiorly; tenderness behind the ear; may be confused with mastoiditis or TMJ pain.	Head rotation, neck strain, tight collars or straps.
VII—Numular/Parietal	Terminal cutaneous branches of occipital or auriculotemporal nerves; small emissary/foraminal vessels; localized subgaleal fascial thickening.	Strictly circumscribed, coin-shaped (2–6 cm) parietal/vertex area with pressure sensitivity and possible allodynia; often chronic and focal.	Direct pressure (helmets, hats), hair manipulation, and weather change.

**Table 3:** PREEMPT Injection Protocol Summary [136].

Muscle	Anatomy Highlights	Dose and Sites	Injection Technique	Key Clinical Notes
Corrugator	Medial superciliary arch; blends with/orbicularis oculi and frontalis; supraorbital and supratrochlear nerves pass through.	5 U × 2 sites (1 each side)	Pinch muscle; inject at 90° with bevel up; feel for muscle pop, inject superficially.	Too superior → brow ptosis; too deep → periosteum pain. Weakening elevates the medial brow.
Procerus	The midline muscle over the nasal bridge; it intermingles with/frontalis and corrugator.	5 U × 1 site	Inject superficially, 90° angle at the midpoint between the corrugator injections.	Too superior → frontalis hit; too deep → periosteum pain.
Frontalis	Thin muscle, elevates eyebrows; supratrochlear and supraorbital nerves pass through.	5 U × 4 sites (20 U total)	Inject the upper 1/3 of the forehead, at least 1–2 fingerbreadths above corrugators; 45° upward angle, superficial.	Too low/lateral → brow ptosis; compensate for preexisting ptosis.
Temporalis	Large fan-shaped muscle; trigeminal motor innervation.	5 U × 8 sites (4 each side → 40 U total)	Use the tragus vertical line; injections at least 2 fingerbreadths above. Administer at 45°.	Prone to bleeding; avoid the superficial temporal artery; repeated injections may cause a scalp “hourglass” appearance.
Occipitalis	Small posterior scalp muscle; near the greater and lesser occipital nerves.	5 U × 6 sites (3 each side → 30 U total)	Inject above the nuchal ridge, at a 45° angle superficially; angle the needle upward away from the neck.	Too low → neck weakness; injections may be painful near the occipital nerves.
Cervical Paraspinal Group	Posterior cervical support muscles; greater/lesser occipital nerves lateral.	5 U × 4 sites (2 each side → 20 U total)	1 cm lateral to midline, 3 cm below inion; second site diagonal upward, 45° angle, superficial.	Too deep/low → neck weakness; keep above nuchal ridge; consider preexisting neck pain.
Trapezius	Large triangular upper back muscle; innervated by the spinal accessory nerve.	5 U × 6 sites (3 each side → 30 U total)	Divide the muscle between the neck line and acromion; inject at midpoints; inject horizontally & superficially.	Too lateral → deltoid injection; too deep/high → shoulder/neck weakness. Pre-assess small-framed patients.

**Table 4:** Overview of recent therapeutic developments in migraine.

Target Class/Therapeutic Approach	Representative Interventions	Therapeutic Mechanism and Benefits	Current Knowledge & Future Perspectives
CGRP monoclonal antibodies	Erenumab, fremanezumab, galcanezumab, eptinezumab	CGRP ligand or receptor blockade reduces trigeminovascular activation and neurogenic inflammation.	Well-established preventive efficacy with favourable tolerability; moving toward first-line therapy. Focus on precision selection and finding biomarkers for predicting responders; needs long-term data in special populations, including paediatrics [229,230,231].
CGRP small-molecule antagonists (gepants)	Rimegepant, ubrogepant, atogepant, zavegepant	Oral/intranasal CGRP receptor antagonism without vasoconstriction.	Effective for acute and preventive treatment, especially in triptan-insufficient responders. Future research includes combination CGRP inhibition and long-term safety [230,232,233].
Ditans (5-HT1F agonists)	Lasmiditan	Trigeminal inhibition through novel selective 5-HT_1F_ activation without vascular effects	Approved for acute treatment of migraine. Option for patients with cardiovascular contraindications. Drawbacks are risk of dizziness, paraesthesia and sedation. Future work defines its position relative to gepants and examines CNS tolerability [234,235,236,237].
PACAP pathway antagonism	Lu AG09222 (anti-PACAP mAb)	Neutralises PACAP, a migraine mediator independent of CGRP with potent trigeminovascular effects.	Phase 2 trials show significant reduction in migraine frequency. PACAP is the leading non-CGRP target; future work needed in CGRP-nonresponsive subtypes [238,239,240].
Peptide/receptor pathways beyond CGRP/PACAP	Amylin/AMY1 antagonists; NK1 modulators	Amylin shares structural and functional overlap with CGRP; neurokinin pathways modulate trigeminal inflammation and sensitization.	Amylin signalling may explain incomplete CGRP response; Potential utility in CGRP-partial responders; renewed interest in next-generation NK1 compounds with adequate CNS penetration [230,240,241,242].
Ion-channel–directed therapies	TRPV1/TRPA1/TRPM8 modulators; KATP blockers; BKCa inhibitors; ASIC inhibitors; TRESK modulation	Regulate neuronal excitability, meningeal nociception, and susceptibility to spreading depolarization, influencing migraine threshold.	High-promise “post-CGRP” frontier. Need selective, safe compounds due to widespread channel expression; possible phenotype-specific therapies (cold-triggered, pressure-sensitive migraines) [228,240,243,244].
Endocrine and hormonal targets	Prolactin receptor antagonists; estrogen stabilization; melatonin agonists	Influence hormonally sensitive trigeminal pathways and cortical excitability.	Highly relevant to menstrual and sex-specific migraine. Future focus on hormone-responsive phenotyping and selective receptor modulation [240,245].
Hypothalamic/sleep–wake regulation pathways	Orexin receptor modulators; neurosteroid/GABAergic agents	Modulate hypothalamic control of sleep, circadian rhythms, metabolism, and nociceptive gain.	Promising for sleep-triggered or chronobiological migraine. Development focuses on selective orexin receptor agents [246,247].
Vascular and metabolic mediators	Nitric oxide pathway modulators; cGMP inhibitors; adenosine receptor ligands	Regulate vascular tone, meningeal perfusion and metabolic sensitivity of trigeminal neurons.	Difficult to target safely due to systemic effects. Future work explores downstream or biased signalling modulators [248,249].
Neuroimmune/inflammatory pathways	P2X7 antagonists; IL-1β/TNF modulators; mast-cell stabilizers	Reduce glial activation, cytokine output, and meningeal inflammatory sensitization.	Particularly relevant to chronic migraine and medication-overuse headache; requires CNS-selective immunomodulation [250,251,252].
Brain-network neuromodulation	Remote electrical neuromodulation (REN), external trigeminal nerve stimulation (eTNS), noninvasive vagus nerve stimulation (nVNS) and single-pulse transcranial magnetic stimulation (sTMS)	External modulation of cortical–brainstem networks and trigeminal pathways.	FDA-cleared non-invasive neuromodulation devices for migraine, with favourable safety profiles; Supported for acute and preventive use. Future work includes closed-loop systems and biomarker-guided neuromodulation [253,254].
Digital therapeutics & virtual care	Digital Cognitive–Behavioral Therapy, remote monitoring, prescription digital therapeutics	Behavior modification, trigger management, and enhanced adherence.	Demonstrated reduction in attack frequency; key tool for access-limited populations. Future priorities include adherence optimisation and digital-equity strategies [253,255,256].
System-level innovations & equity-focused care	Telehealth, primary-care algorithms, managed-care optimization	Aims to address underdiagnosis, delays in treatment, and disparities among socioeconomically, racially, and geographically diverse groups.	Critical to reduce underdiagnosis and undertreatment. Emphasis on systematic screening, education, and equitable deployment of novel therapies [253,255,257].
Precision-medicine and biomarker development	Genomic panels, neuropeptide profiling, imaging phenotypes	Matches patients to mechanism-appropriate therapy (CGRP vs. PACAP vs. ion-channel vs. neuromodulation).	Considered a critical future direction; essential for moving migraine management toward a personalized approach [230,240].
Photobiomodulation-based interventions (peripheral and intravascular laser therapies)	-Low-level laser therapy (LLLT): Infrared diode laser, 808 nm, 100 mW, 120 J/cm^2^, applied to 31 cranial/cervical trigger-point sites in 10 sessions over one month [246]. -Intravascular laser irradiation of blood (ILIB): He–Ne laser, 632.8 nm, 1–5 mW/cm^2^ delivered intravenously over 10 daily sessions [247]	LLLT (peripheral photobiomodulation): Proposed modulation of nociceptive input through effects on peripheral trigger points, reduction in muscular hyperactivity, local anti-inflammatory and photobiomodulatory effects influencing ATP, NO, and oxidative pathways [258]. ILIB (systemic photobiomodulation): Augmentation of micro- and macrovascular perfusion, enhancement of regional cerebral blood flow, modulation of serotonergic pathways, and systemic anti-inflammatory effects [259].	LLLT: In a randomized open-label study of 36 chronic migraine patients, LLLT demonstrated significant reductions in headache days, pain intensity and acute medication use, with efficacy comparable to onabotulinumtoxinA. Improvements in sleep disturbances favoured LLLT, although the trial was small, unblinded, and short-term [258]. ILIB: In a 24-patient observational study, ILIB significantly improved pain scores, migraine-related disability, sleep quality, cognition and increased regional cerebral blood flow on SPECT imaging. Effects persisted at one month. Evidence remains preliminary and non-randomized [259]. In perspective, photobiomodulation may represent a non-pharmacologic adjunct targeting neurovascular dysfunction in migraine. However, heterogeneity in wavelengths, dosimetry, anatomical targeting, and trial design need future analysis

## Data Availability

No new data were created.
